# The trehalose 6-phosphate pathway coordinates dynamic changes at the shoot apical meristem in *Arabidopsis thaliana*

**DOI:** 10.1093/plphys/kiaf300

**Published:** 2025-07-10

**Authors:** Magdalena Musialak-Lange, Katharina Fiddeke, Annika Franke, Friedrich Kragler, Christin Abel, Vanessa Wahl

**Affiliations:** Department of Metabolic Networks, Max Planck Institute of Molecular Plant Physiology, Potsdam 14476, Germany; Department of Metabolic Networks, Max Planck Institute of Molecular Plant Physiology, Potsdam 14476, Germany; Department of Metabolic Networks, Max Planck Institute of Molecular Plant Physiology, Potsdam 14476, Germany; Department of Metabolic Networks, Max Planck Institute of Molecular Plant Physiology, Potsdam 14476, Germany; Department of Metabolic Networks, Max Planck Institute of Molecular Plant Physiology, Potsdam 14476, Germany; Department of Metabolic Networks, Max Planck Institute of Molecular Plant Physiology, Potsdam 14476, Germany

## Abstract

A plant's stem cell population in the shoot apical meristem (SAM) is maintained by WUSCHEL (WUS) and CLAVATA3 (CLV3). SAM size is dynamic and undergoes a more than 2-fold expansion upon transition to reproductive growth. The mechanism controlling this doming is largely unknown; however, coinciding increased trehalose 6-phosphate (T6P) levels suggest a participation of the T6P pathway in Arabidopsis (*Arabidopsis thaliana*). Moreover, lines misexpressing or with reduced expression of *TREHALOSE PHOSPHATE SYNTHASE1* (*TPS1*) have smaller and larger SAMs, respectively. Here, we show that *TREHALOSE PHOSPHATE PHOSPHATASEJ* (*TPPJ*) is directly regulated by WUS. Changing *TPPJ* transcript levels in the outer layer affects SAM size and flowering time, and its reduction in the late-flowering *clv3* mutant restores wild-type flowering. This is associated with altered mature microRNA156 abundance and expression of the *SQUAMOSA PROMOTER-BINDING PROTEIN-LIKE* genes *SPL3*, *SPL4*, *SPL5*, and *SPL9*. Furthermore, *SPL4* is controlled by WUS, while SPL4 directly represses *WUS*, establishing negative feedback regulation. This feedback loop is important for age pathway-induced flowering involving the T6P pathway and suggests dynamic feedback regulations between central meristem maintenance and flowering time regulators with sugar signaling throughout development.

## Teaser

An identified feedback loop demonstrates dynamic feedback regulations between central meristem maintenance and flowering regulators with trehalose 6-phosphate signaling.

## Introduction

All above-ground plant organs such as leaves and flowers originate from the shoot apical meristem (SAM). The Arabidopsis (*Arabidopsis thaliana*) SAM is organized in 3 clonally distinct cell layers: the outer single cell layers, L1 and L2, and the multiple cell files comprising L3. Functionally, the SAM consist of a central zone (CZ), containing undifferentiated cells with a self-renewing potential, an organizing center (OC), inducing and maintaining an adequate number of cells in the CZ, a peripheral zone (PZ), which receives cells from the CZ that are differentiating and incorporated into leaf or flower primordia, and a rib zone, producing the ground tissue and the inflorescence stem (Pfeiffer et al. 2017). Communicating position-dependent properties among cells within and between the different zones is crucial for SAM function. Two noncell autonomously acting factors regulate each other in a negative feedback loop. The homeodomain transcription factor *WUSCHEL* (*WUS*) is expressed in the OC, and the signaling peptide *CLAVATA3* (*CLV3*) in the CZ. Thus, mobile WUS transcription factor, secreted from the OC, activates expression of *CLV3* in the CZ above, while CLV3 peptide restricts the *WUS* expression domain to the OC. Hence, *WUS* and *CLV3* expression domains are considered mutually exclusive and their negative feedback regulation as very robust, which can only be modulated within tight limits (Pfeiffer et al. 2017).

Development as a whole and SAM maintenance in particular demand continuous crosstalk between its regulatory processes and the available resources. The first sign of the floral transition to occur in many plant species is an increase in the rate of cell division at the SAM causing a dramatic increase in height relative to width, changing it from a rather flat to a domed shape. This process is known as doming (Bernier 1998; Jacqmard et al. 2003; Kwiatkowska 2008) and marks the transition between the vegetative (production of leaves) and the reproductive stage (formation of flowers). A study in tomato, but also recent work in Arabidopsis, demonstrated that a doming wild-type SAM is associated with an increased *WUS* domain, but also indicated other factors acting in parallel (Tal et al. 2017; Bertran Garcia de Olalla et al. 2024). This supports a previously suggested multifactorial control of the process (Bernier 1998). On the same note, only recently early, cellular changes in Arabidopsis were associated with GA biosynthesis (Kinoshita et al. 2020) and APETALA2 (Bertran Garcia de Olalla et al. 2024). However, knowledge on the mechanisms controlling doming is still scarce.

Since floral transition requires a massive reorganization of organ development and sufficient energy, it is tightly controlled by environmental conditions (Srikanth and Schmid 2011) and availability of nutrients (Wahl et al. 2013; Olas et al. 2019; Gramma et al. 2024). In plants, the sucrose signal trehalose 6–phosphate (T6P) is synthesized from UDP-glucose and glucose 6-phosphate via TREHALOSE PHOSPHATE SYNTHASE (TPS) and is dephosphorylated to trehalose by TREHALOSE PHOSPHATE PHOSPHATASE (TPP) (Fichtner and Lunn 2021). TPS proteins appear in 2 classes. Class 2 TPSs have recently been shown to interact with and negatively regulate the central metabolic regulator SnRK1 (Van Leene et al. 2022), although in contrast to Class 1 TPSs, such as the Arabidopsis TPS1, they are not catalytically active in yeast (Delorge et al. 2015). Similar to Class 1 TPSs, the 10 Arabidopsis TPP proteins are catalytically active in yeast, indicating that they can convert T6P to trehalose in planta (Vandesteene et al. 2012). T6P serves as a local signal for sucrose availability, which is conveyed to downstream metabolic and growth responses through still largely unknown mechanisms. Cell-to-cell movement, a prerequisite for long-distance T6P signaling, has been proposed but not demonstrated so far (Fichtner and Lunn 2021). In Arabidopsis, sugar signaling affects the vegetative phase change, the transition between juvenile and adult vegetative stage, via the T6P pathway (Yang et al. 2013; Yu et al. 2013; Ponnu et al. 2020). In addition, the T6P pathway is involved in shoot branching (Fichtner et al. 2020) and induces flowering via regulating key flowering genes in leaves and at the SAM (Wahl et al. 2013). In leaves, *TPS1* is necessary and sufficient to induce *FLOWERING LOCUS T* (*FT*), the florigen, connecting a photoperiod signal with a physiological signal. In addition, the T6P pathway affects the age-dependent microRNA156 (miR156)/*SQUAMOSA PROMOTER-BINDING PROTEIN-LIKE* (*SPL*) module at the SAM (Wahl et al. 2013). Increased T6P levels coincide with doming (Wahl et al. 2013), and as we show now, a spatial change of the *WUS* expression domain. This suggests a participation of sugar signaling in regulating SAM dynamics through transient uncoupling of the negative feedback regulation between WUS and CLV3. Here, we demonstrate that WUS directly regulates *TREHALOSE PHOSPHATE PHOSPHATASEJ* (*TPPJ*). Decreased expression of *SPL* genes and increased abundance of mature miR156 cause a late-flowering phenotype of *clv3*. This phenotype is restored when *TPPJ* is downregulated in the outer meristem layer. We further demonstrate a negative feedback loop between WUS and SPL4 downstream of the T6P pathway. In summary, we provide evidence for a dynamic regulation of central meristem maintenance and flowering time regulators that involves the T6P pathway.

## Results

### The T6P pathway affects SAM size

**Figure 1.**
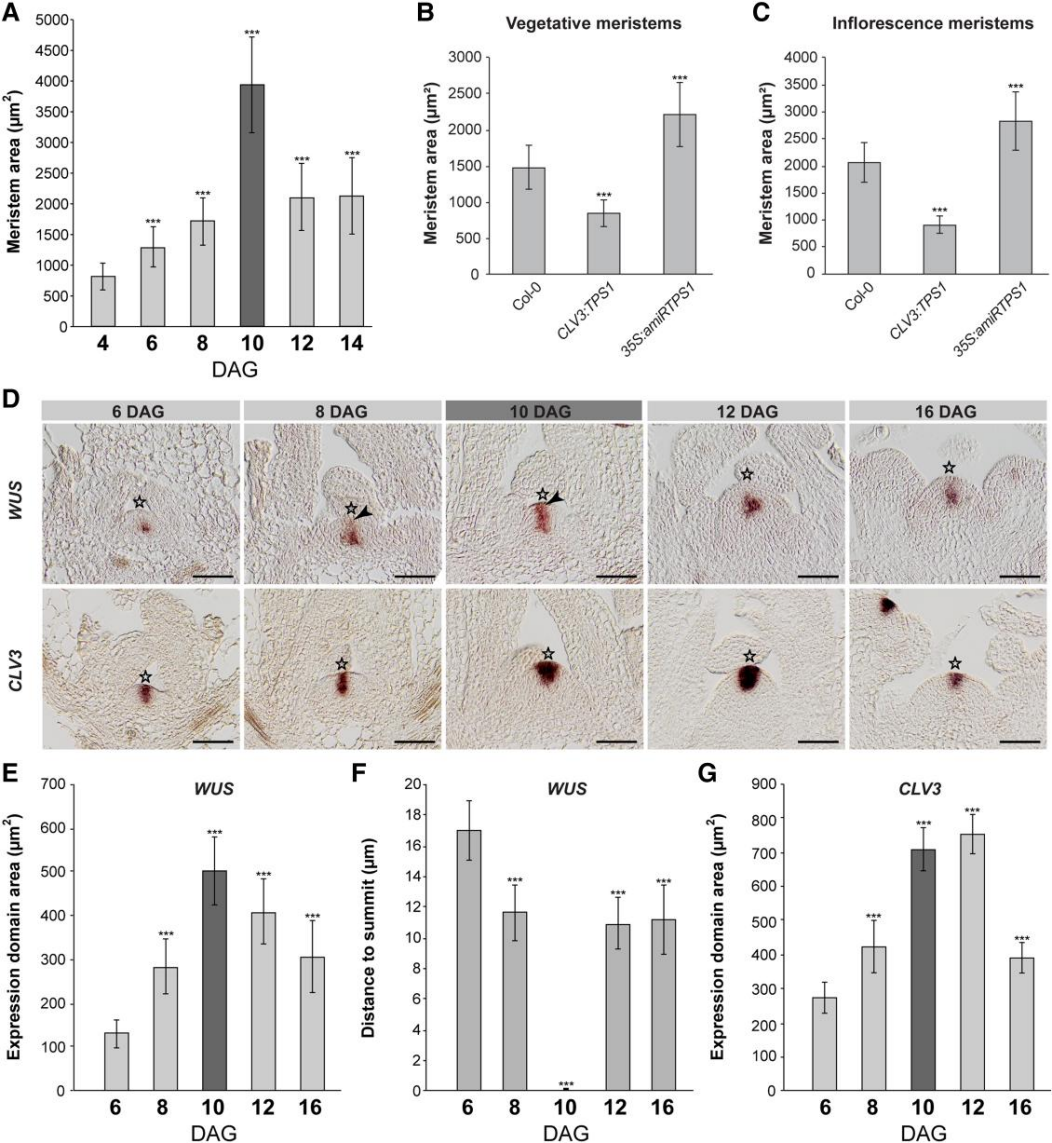
The T6P pathway impacts Arabidopsis SAM size during development. **A)** SAM area throughout development. *n* = 15 per time point. **B)** Vegetative and **C)** inflorescence SAM size of *CLV3:TPS1* and *35S:amiRTPS1* lines. *n* = 10 per genotype and time point. **D)**  *WUS* and *CLV3* expression by RNA in situ hybridization in vegetative (6 and 8 DAG), transition (10 DAG, marked dark gray) and inflorescence SAMs (12 and 16 DAG) of LD-grown Col–0 plants. Arrowhead indicates *WUS* expression in outer SAM layer. **E)**  *WUS* expression domain sizes, **F)**  *WUS* expression domain distance to SAM summit, and **G)**  *CLV3* expression domain area, in vegetative, transition (dark gray) and inflorescence SAMs of LD-grown wild-type plants. *n* > 10 per time point. Error bars denote Sd; significance was calculated based on a Student's *t*-test, ***P < 0.001. Star indicates SAM summit. Scale bars are 25 *µ*m.

To assess whether the morphological changes at the transition SAM (Fig. 1A) involve the T6P pathway, we investigated SAM architecture upon decreasing *TPS1* by the means of an artificial microRNA in the SAM proper (*35S:amiRTPS1*; Supplementary Fig. S1) and increasing *TPS1* in the CZ (*CLV3:TPS1*; Supplementary Fig. S2). These plants have smaller and bigger vegetative and reproductive meristems (Fig. 1, B and C), resulting in smaller and larger plants, respectively (Wahl et al. 2013).

*TPS1* knockout mutants are embryo lethal, likely as a result of insufficient cell cycle activity at torpedo stage of embryogenesis (Gómez et al. 2006). Cell cycle genes were also found to be highly induced at transition to flowering (Jacqmard et al. 2003; Klepikova et al. 2015). In line with these findings, the floral transition coincides with increased T6P levels (Wahl et al. 2013). Throughout the vegetative phase, SAM size gradually increases due to rising cell numbers (Fig. 1A; Supplementary Fig. S3) (Kinoshita et al. 2020), reaching its maximum at floral transition, independent of whether the plants are grown continuously in long days (LD) or short days (SD) (Fig. 1A) or in SD followed by a transfer to LD (Supplementary Fig. S4A) (Olas et al. 2019).

### Spatial changes of *WUS* expression in a transition SAM

To assess whether floral transition coincides with effects on meristem maintenance and is thus related to changes of *WUS* or *CLV3* expression in a wild-type SAM similar to what has been demonstrated in tomato (Tal et al. 2017), we analyzed a time series spanning floral transition (Fig. 1D). We found that in vegetative and reproductive SAMs, *WUS* is expressed in the OC and *CLV3* in the CZ. However, a larger SAM at floral transition (in LD and SD) associates with an enlarged *WUS* expression domain; though, unlike in tomato, it expands into the CZ including the outer cell layer (L1) (Fig. 1, D to F; Supplementary Fig. S4B).

Cytokinin signaling has been reported to respond to carbon in seedlings (Pfeiffer et al. 2016; Snipes et al. 2018). Its transient occurrence in L1 may thus explain the presence of *WUS* transcript in the outer SAM layers. However, cytokinin levels are not altered in L1 cells as indicated by the synthetic cytokinin reporter *TCSn:GFP* (Zurcher et al. 2013) (Supplementary Fig. S5). Furthermore, while *WUS* expands to L1 for a short period (8 to 10 d after germination [DAG]), the *CLV3* expression domain enlarges at floral transition (10 DAG) but remains expanded in a young inflorescence SAM (12 DAG), when *WUS* is confined to the OC again (Fig. 1, D and G). This suggests a transient uncoupling of the negative feedback regulation between WUS and CLV3, which is fully re-established at the reproductive SAM (16 DAG), resembling earlier vegetative SAM expression patterns (6 DAG) (Fig. 1D).

### *TPPJ* is directly regulated by WUS

**Figure 2.**
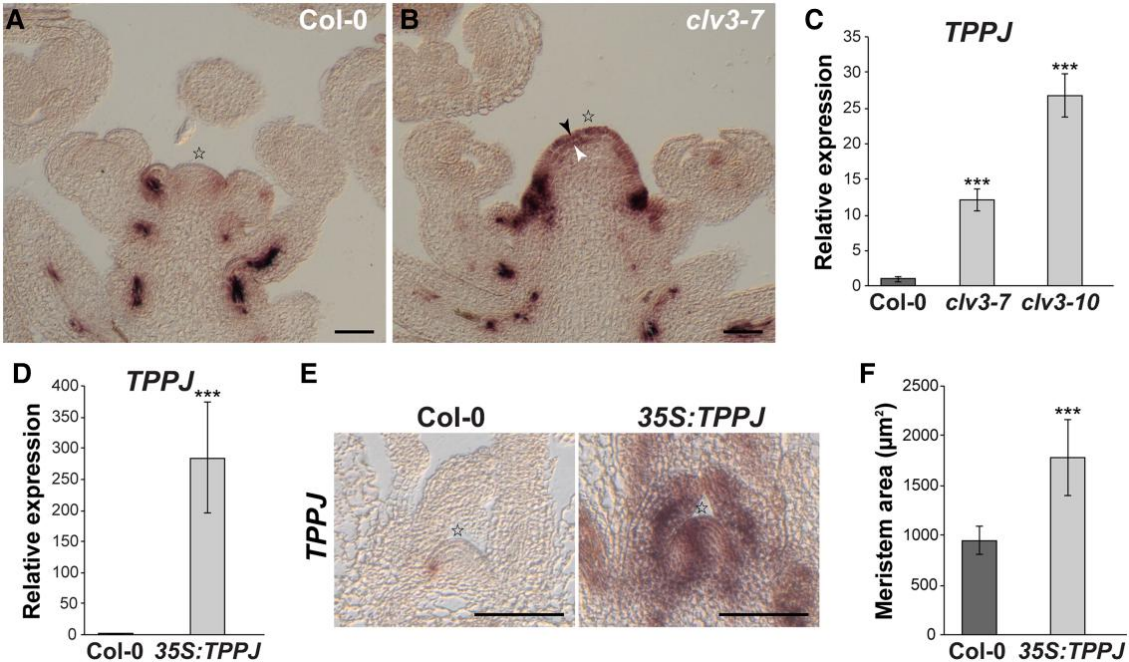
*TPPJ* plays a role at the Arabidopsis SAM. **A**, **B)**  *TPPJ* expression by RNA in situ hybridization on longitudinal sections through inflorescence SAMs of **A)** Col-0 and **B)**  *clv3-7* and by RT-qPCR in apices collected from **C)**  *clv3-7*, *clv3-10*, and **D)**  *35S:TPPJ* plants. Each *n* = 3 per genotype. **E)** Expression of *TPPJ* by RNA in situ hybridization on longitudinal sections through inflorescence SAMs of Col-0 and *35S:TPPJ.*  **F)** SAM size of plants overexpressing *TPPJ*. *n* = 10 per genotype. Error bars denote Sd; significance calculated by 1-way ANOVA **C**, **D)** and Student's *t*-test **F)**, ***P < 0.001. Black and white arrowheads indicate first and second meristem layers, respectively. Stars indicate SAM summit. Scale bars are 50 *µ*m.

To understand how the T6P pathway might control the WUS/CLV feedback loop at the floral transition, we looked into the expression pattern of the 10 genes encoding TPPs in Arabidopsis. Reporter lines for each of these genes using a 2-kb upstream sequence coupled to GUS have previously revealed complex spatiotemporal patterns at distinct developmental stages in diverse organs including roots, leaves, flowers, and seeds, suggesting tissue-specific effects on T6P abundance (Vandesteene et al. 2012). However, this data set did not comprise sections through the SAM. We therefore made use of RNA in situ hybridization and specific *TPP* probes to analyze the cellular resolution of their expression on tissue sections through vegetative and inflorescence apices (Supplementary Fig. S6). We found that *TPP* genes are expressed in very distinct SAM domains in a stage-dependent manner (Supplementary Fig. S6). This excludes *TPPC* and *TPPD*, which are expressed below detection limit in adult plant apices, which had been suggested before (Vandesteene et al. 2012). Remarkably, *TPPJ* strongly increases in the enlarged SAM of *clv3–7* due to an ectopic expression in L1 and L2 (Fig. 2, A to C; Supplementary Figs. S7 and S8). Notably, ectopic expression of *TPPJ* (Supplementary Fig. S8) in *clv3-7* and *clv3-10* coincide with high levels of *WUS* in the outer SAM layers (Brand et al. 2000). In contrast, *TPPJ* is repressed in a *wus-7* background (Supplementary Fig. S9A). In addition, a published data set shows induced levels of *TPPJ* upon DEX-induced expression of *WUS* in *35S:WUS-GR* plants (Busch et al. 2010) (Supplementary Fig. S9B).

To assess whether ectopic expression of *TPPJ* might contribute to the enlarged SAM of the *clv3* mutant, we overexpressed *TPPJ* in the wild type (*35S:TPPJ*; Fig. 2, D and E). This resulted in plants with significantly larger SAMs (Fig. 2F).

**Figure 3.**
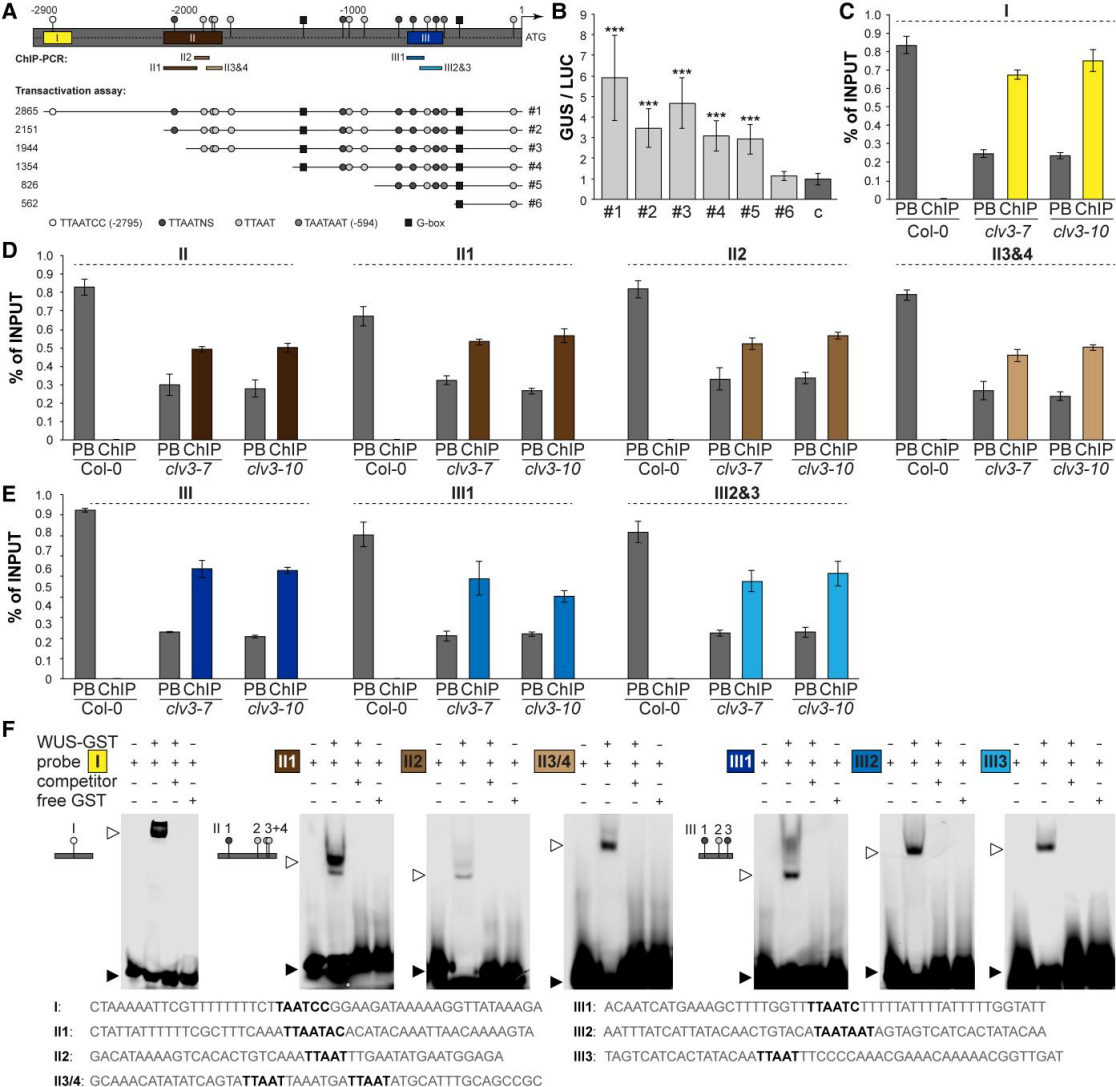
WUS directly regulates *TPPJ* in the Arabidopsis SAM. **A)** Overview of *TPPJ* 5′ regulatory region with putative *TPPJ^WUS^* sites (gray circles, black boxes), position of ChIP-PCR amplicons corresponding to the results shown in **C)** to **E)**. Boxes marked with I, II, and III indicate *5′ TPPJ* regions with in total 7 confirmed core *TPPJ^WUS^* sites—I: −2,795 to −2,789 bp, II: −2,073 to −1,830 bp, and III: −652 to −564 bp. Sequence location and lengths used in **B)** are indicated with #1 to 6. **B)** Protoplast transactivation assay showing activation of the GUS reporter when coupled to the regions indicated in **A)**, relative to LUC activity. c indicates untransformed control. *n* = 6. **C** to **E)** Enrichment of **C)** Region I, **D)** Region II, and **E)** Region III as indicated in **A)** measured by ChIP–PCR relative to the input. PB, postbinding fraction. *n* = 3. **F)** EMSA for WUS binding to the indicated regions (**A**, I to III). Note the shifted band in the presence of WUS protein (open arrowhead) and the nonshifted fraction (closed arrowhead). Error bars denote Sd; significance based on 1-way ANOVA, ***P < 0.001.

These results imply a direct influence of WUS on *TPPJ* and are supported by an in silico analysis, which predicts multiple, canonical WUS-binding sites in the sequence upstream of *TPPJ* (*TPPJ*^WUS^; Fig. 3A) (Lohmann et al. 2001; Leibfried et al. 2005; Yadav et al. 2011; Sloan et al. 2020). To understand if WUS directly controls *TPPJ*, we performed in vivo transactivation assays. These show that WUS activates reporter gene expression when using a 2,865-bp 5′*TPPJ* sequence, containing 17 putative *TPPJ*^WUS^ sites (Fig. 3A). Progressive deletion of the sequence resulted in a reduction of reporter gene activation, suggesting an additive effect of the individual *TPPJ*^WUS^ sites (#1 to 6; Fig. 3, A and B). We confirmed direct binding of WUS to 3 distinct regions of the *TPPJ* promoter (I, II, and III; Fig. 3, C to E; Supplementary Fig. S10) by chromatin immunoprecipitation (ChIP) coupled to quantitative PCR (qPCR) using a specific antibody against WUS (Supplementary Figs. S10 and S11), while all the other regions did not indicate binding (Supplementary Figs. S10 and S12). We observed enrichment of WUS binding to *TPPJ*^WUS^ sites up to 0.67% of the input DNA in *clv3-7* and 0.75% of the input DNA in *clv3-10* apices, both of which express *WUS* at very high levels in comparison to input DNA from wild-type apices, where *WUS* is expressed in only a few cells and input DNA from leaves, with no WUS (Figs. 3, C to E; Supplementary Fig. S10). In electrophoretic mobility shift assays (EMSA), we observed a specific shift of all bands in the presence of WUS, which was abolished in the presence of a specific competitor. Additionally, mutation in the core WUS-binding site of the competitor sequence resulted in a shift, confirming specific in vitro binding of WUS to the investigated sequences (I, II 1 to 3, and III 1 to 3; Fig. 3F; Supplementary Fig. S13). In summary, we demonstrate that 7 out of 17 putative *TPPJ*^WUS^ sites are directly targeted by WUS in vivo.

### TPPJ controls flowering and SAM size from the outer layer

A *tppj* mutant with a T-DNA insertion in the sixth exon, which knocks out *TPPJ* expression with neglectable effect on the expression of the other *TPP* genes (Supplementary Fig. S14), flowers significantly earlier than the wild type (Supplementary Table S1). Due to the prominent input of the photoperiod pathway, the effect was much milder in LD (Supplementary Table S1). To further assess the role of *TPPJ* at the SAM, we used an artificial miRNA (amiR) approach (Schwab et al. 2006) to specifically downregulate *TPPJ* without any off-target effects on the expression of the other *TPP* genes (*35S:amiRTPPJ*; Supplementary Fig. S15). Plants overexpressing either of 2 versions of an *amiRTPPJ* (V1 and V2) flower significantly earlier in LD and SD (Supplementary Fig. S16 and Table S1).

**Figure 4.**
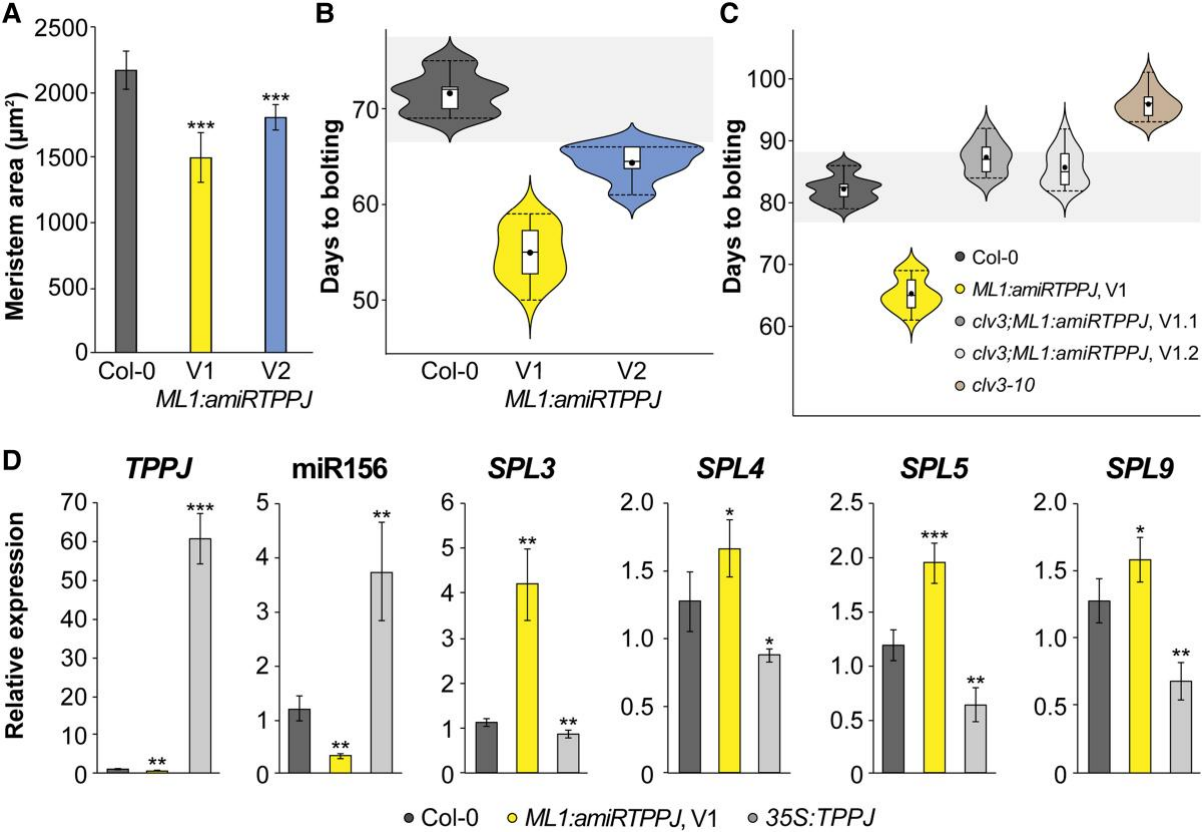
The role of TPPJ in the outer Arabidopsis SAM layer. **A)** Meristem area of Col-0, *ML1:amiRTPPJ* V1 and V2. *n* = 10 per genotype. **B**, **C)** Flowering time of **B)**  *ML1:amiRTPPJ* and **C)**  *clv3-10;ML1:amiRTPPJ* shown as days to bolting relative to Col-0. *n* = 22 per genotype. V1 and V2 indicate 2 independent versions of artificial microRNAs designed to target *TPPJ* transcript. The whiskers indicate the highest and lowest values, the box indicates the interquartile range including the upper and lower quartiles, the center line indicates the median, the black dots indicate the mean, and the width indicates the abundance of values. **D)** Relative expression of *SPL* genes in SD-grown *ML1:amiRTPPJ* and *35S:TPPJ* at 40 DAG. *n* = 4 per genotype. Error bars denote Sd; significance calculated based on 1-way ANOVA **D)** and Student's *t-*test **A)**; *P < 0.05, **P < 0.01, and ***P < 0.001.

*MERISTEM LAYER1* (*ML1*) expression specifically and stably localizes to L1 in all investigated stages (Supplementary Fig. S17A). *ML1:TPPJ* plants have a larger SAM as compared to wild type (Supplementary Fig. S17B). Furthermore, *ML1:amiRTPPJ* reduces *TPPJ* expression in L1 (Supplementary Fig. S17C), has a smaller SAM, similar to *tppj* plants (Supplementary Fig. S17D), and flowers significantly earlier in LD and SD (Figs. 4, A and B; Supplementary Fig. S17E and Table S1), while the level of *TPPJ* reduction is proportional to the acceleration of flowering (Figs. 4, B and C; Supplementary Fig. S15 and Table S1).

To date, there are no reports that mutants in meristem maintenance genes have a flowering phenotype. Surprisingly, we found that *clv3* plants are late flowering (Fig. 4C; Supplementary Table S1). However, when *ML1:amiRTPPJ* is introgressed into the *clv3–7* or *clv3–10* background, the late-flowering phenotype is restored to wild type (Fig. 4C; Supplementary Fig. S18 and Table S1), suggesting that *TPPJ* expression in the outer meristem layers is causal for the late-flowering phenotype of *clv3*. Hence, the early flowering of *ML1:amiRTPPJ* in a wild-type background is due to a reduction of *TPPJ* expression in the outer meristem layer in its endogenous expression domain. In addition, other prominent morphological defects of *clv3–10* such as the fasciated stem and enormous inflorescence SAM are visibly reduced when *ML1:amiRTPPJ* was introgressed into *clv3* (Supplementary Fig. S18).

At floral transition, an increasing *WUS* expression domain through the uncoupling from CLV3 regulation expands into the CZ and the outer cell layer (L1) results in an enlarging SAM (Fig. 1, D to F). It is important to note that *TPPJ* overexpression also leads to significantly enlarged SAMs (Fig. 2F). This is likely due to the T6P pathway's impact on *WUS* or *CLV3* (Supplementary Fig. S19, A to C). Interestingly, both *WUS* and *CLV3* expressions increase to much higher levels in *ML1:amiRTPPJ* (Supplementary Fig. S19, B and C). Importantly, a larger OC domain marked by *WUS* (Supplementary Fig. S19A) overlaps with the stem cell pool, marked by *CLV3* (Supplementary Fig. S19A) when *TPS1* levels are reduced (*35S:amiRTPS1*), while stem cell pool size increases with little effect on *WUS* when plants overexpress *TPS1* (*35S:TPS1*; Supplementary Fig. S19A). This suggests an active role of the T6P pathway regarding stem cell maintenance. Furthermore, CZ size decreases when *TPS1* is expressed directly into the stem cell niche from the *CLV3* promoter (Supplementary Fig. S19A), in support of a much smaller SAM of the very early flowering *CLV3:TPS1* (Wahl et al. 2013), indicating that fine-tuning between the regulatory pathways is crucial to maintain the stem cell number.

### WUS regulates the age pathway through *SPL4*

**Figure 5.**
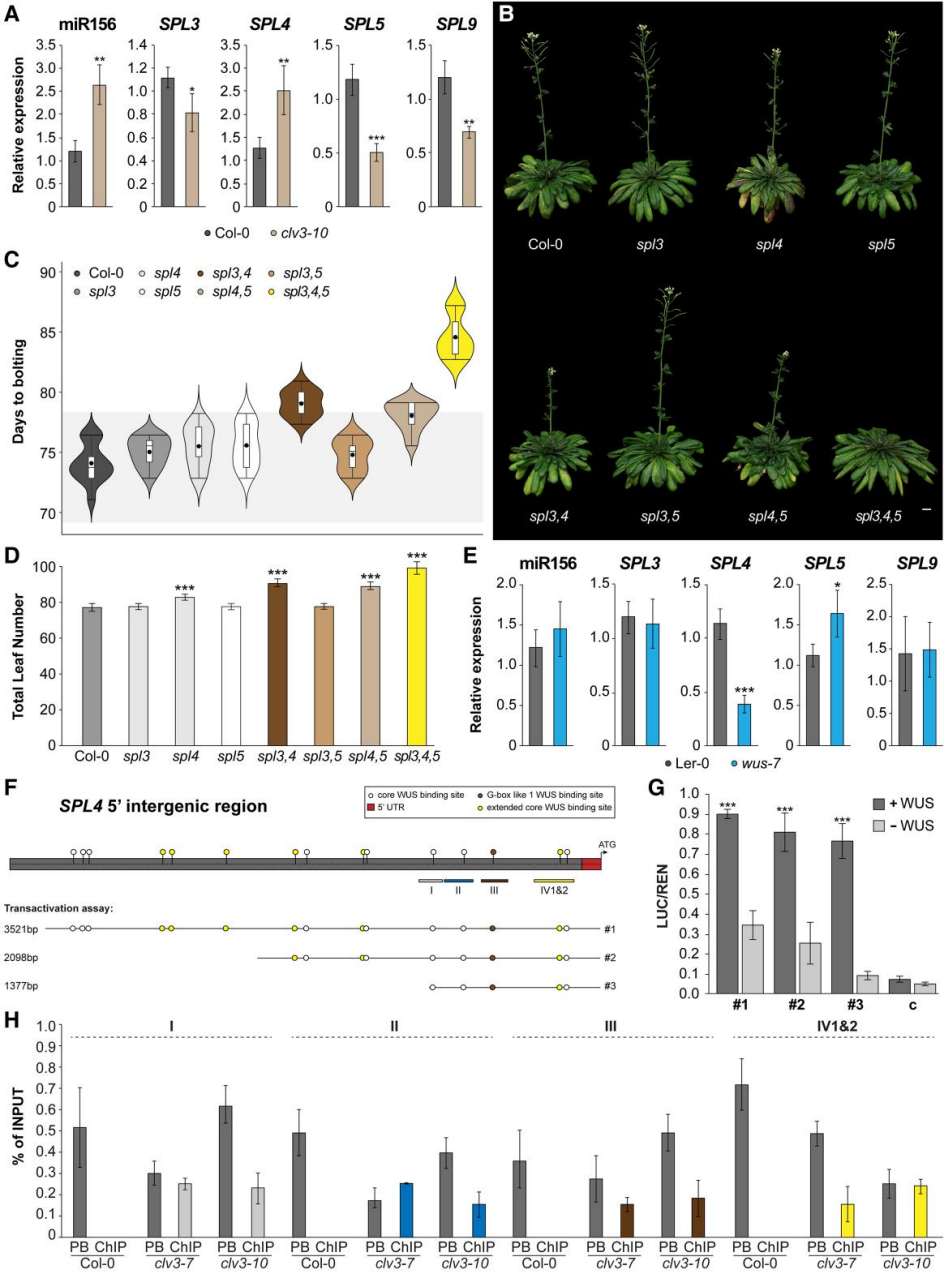
WUS activates *SPL4* in Arabidopsis. **A)** Relative expression of mature miRNA and *SPL* genes in SD-grown Col-0 and *clv3-10* apices. *n* = 4 per genotype. **B)** Representative pictures of *SPL3-5* single, double, and triple CRISPR/*Cas9* deletion mutants in comparison to Col-0 grown in SD. Images were digitally extracted for comparison. Scale bar is 1 cm. **C)** Flowering time of deletion mutants displayed in **B)** provided as days to bolting and **D)** total leaf numbers. *n* = 25 per genotype. The whiskers indicate the highest and lowest values, the box indicates the interquartile range including the upper and lower quartiles, the center line indicates the median, the black dots indicate the mean, and the width indicates the abundance of values. **E)** Relative expression of mature miRNA and *SPL* genes in SD-grown L*er*-0 and *wus-7* apices. *n* = 3 per genotype. **F)** Overview of *SPL4* 5′ regulatory region with putative *SPL4^WUS^* sites (indicated as circles) and position of ChIP-PCR amplicons (indicated as boxes below sequence) corresponding to the results shown in **G)** and **H)**. Boxes marked with I, II, III, and IV (1&2) indicate *5′ SPL4* regions with in total 5 core *SPL4^WUS^* sites—I: −1,073 to −1,068 bp, II: −880 to −875 bp, III: −697 to −691, IV1: −259 to −252, and IV2: −214 to −209 bp. Sequence location and lengths used in **B)** are indicated with #1 to 3. **G)** Protoplast transactivation assay showing activation of the reporter (LUC) when coupled to the regions indicated in **F)**, relative to REN activity. c indicates the vector control. *n* = 3. **H)** Enrichment of Regions I, II, III, and IV1&2 as indicated in **F)** measured by ChIP–PCR relative to the input in *clv3–7* and *clv3-10* apices. *n* = 3. LUC, luciferase; PB, postbinding fraction; REN, renilla. Error bars denote Sd; significance calculated based on 1-way ANOVA **A**, **E)** and Student's *t-*test **D**, **G)**; *P < 0.05, **P < 0.01, and ***P < 0.001.

Fouracre and Poethig (2019) indicated an active pool of miR156 in the SAM, important for early shoot maturation, and we previously reported that the T6P pathway influences the age pathway at the SAM (Wahl et al. 2013). We therefore next analyzed mature miR156, as well as the expression of the miR156 targets *SPL3*, *SPL4*, *SPL5*, *SPL9*, and *SPL15*, all associated with floral transition (Hyun et al. 2017; Xie et al. 2020). We found a strong reduction of mature miR156 levels associating with decreased *TPPJ* in *ML1:amiRTPPJ*. Correspondingly, we found increased expression of *SPL3*, *SPL4*, *SPL5*, and *SPL9* (Fig. 4D). In line, miR156 was more abundant in *35S:TPPJ*, while the corresponding *SPL*s were downregulated (Fig. 4D). Expression of *SPL15*, previously described as an important integrator of plant age into the regulation of floral onset in SD (Wang et al. 2009; Yamaguchi et al. 2014; Hyun et al. 2016), was not differentially expressed in the transgenic lines (Supplementary Fig. S20A). Importantly, we also found miR156 levels significantly increased in *clv3–10* apices (Fig. 5A). In response, *SPL3*, *SPL5*, and *SPL9* expression was reduced supporting the late-flowering phenotype of the mutant, while *SPL15* was not affected (Supplementary Fig. S20B). This suggests that uncoupling of WUS from CLV3 and expansion of its expression domain occurs downstream of the T6P pathway and involves age pathway components. In contrast to *SPL3*, *SPL5*, and *SPL9* expression, we found increased *SPL4* transcript in the apices of the late-flowering *clv3–10* (Fig. 5A; Supplementary Fig. S21).

The contribution of SPL9 and SPL15 to the regulation of flowering is well established (Wang et al. 2009; Yamaguchi et al. 2014; Hyun et al. 2016), but there is some dispute as to whether SPL3, SPL4, and SPL5 are important. While overexpression of *SPL3*, *SPL4*, and *SPL5* induces flowering (Wu and Poethig 2006; Jung et al. 2016), a triple T-DNA insertion/tilling mutant was reported to not display any flowering phenotype (Xu et al. 2016). However, a recent study analyzed a triple deletion mutant generated by CRISPR/*Cas9* in relation to light signaling and also reported a delay in flowering time in LD (Xie et al. 2020). This line flowered significantly later in our SD conditions (Supplementary Table S1), confirming the earlier observation in LD (Xie et al. 2020). Independently, we generated individual, double and triple deletion mutants in the respective SPLs using CRISPR/*Cas9* (Supplementary Fig. S22). First, our *spl345* line flowered over 10 d later in SD conditions (Supplementary Table S1; Fig. 5C). Second, of the single *spl* mutants, only *spl4* was slightly but significantly later flowering both based on the number of days to bolting and the number of total leaves at flowering (Fig. 5, C and D). Lastly, higher order *spl3* and *spl5* deletion mutants flowered significantly later in all lines with *spl4* (Fig. 5, B to D; Supplementary Table S1). This argues for an important role of *SPL4* in inducing flowering. *SPL4*, similar to *SPL3* and *SPL5*, is not expressed during the vegetative stage but induced at floral transition at the wild-type SAM (Schmid et al. 2003). It is expressed in the center of the SAM, in a domain overlapping with the cells containing the highest levels of WUS, while *SPL3*, *SPL5*, *SPL9*, and *SPL15* are expressed at the periphery of the SAM and in the vasculature of young leaves (Wang et al. 2009; Yadav et al. 2011; Daum et al. 2014; Hyun et al. 2016; Olas et al. 2019). This denotes a direct regulation of *SPL4* by WUS, which would explain increased *SPL4* in the *clv3* mutant (Fig. 5A). Indeed, we observed reduced expression of *SPL4* in *wus-7* apices. In contrast, none of the other *SPL*s were downregulated (Figs. 5E; Supplementary Fig. S20C) and miR156 levels did not change (Fig. 5E).

We identified a larger number of potential *SPL4*^WUS^ sites when we compared its 5′ regulatory sequence to that of the other *SPL*s (Fig. 5F; Supplementary Fig. S23). The 15 putative *SPL4*^WUS^ sites are distributed between −3,399 and −217 bp from the transcription start site (Fig. 5F). A transactivation assay using three 5′*SPL4* fragments progressively shortened from the 5′ end (#1: 3,521 bp, #2: 2,398 bp, and #3: 1,377 bp) demonstrated induction of the luciferase reporter by WUS, with the shortest fragment covering most of the inductive effect (Fig. 5G). This sequence contains five *SPL4*^WUS^ sites, four of which could be separately tested by ChIP-qPCR. We observed substantial enrichment of WUS binding to *SPL4*^WUS^ sites of up to 0.25% of the input DNA in *clv3-7* and 0.24% of the input DNA in *clv3-10* apices, confirming direct binding of *SPL4* by WUS (Figs. 5H; Supplementary Fig. S24). However, taken together, these results also suggest that additional players are important for the onset of flowering downstream of the WUS/CLV3 feedback loop, which cannot be bypassed by an otherwise inductive SPL4 in *clv3* mutant plants (Fig. 5A; Supplementary Fig. S21).

### Transient feedback regulation between WUS and the age pathway at floral transition

We further investigated whether the doming process is in part affected by the age pathway downstream of the T6P pathway. While we found *WUS* to be generally induced in all transition SAMs, its expression in *spl3,4* SAMs was significantly higher than in a wild-type SAM (Supplementary Fig. S25, A and B). However, while the WUS domain stretches to the outer meristem layer in a wild-type and *spl3,5* SAM, this does not seem to be the case in SAMs of *spl3,4*, *spl4,5*, and *spl3,4,5* plants (Supplementary Fig. S25A). This cannot be explained by an altered expression of *CLV3*, as, while generally induced at transition in all lines, its expression tends to rather be reduced in all mutant lines when compared to the wild type (Supplementary Fig. S25, C and D).

**Figure 6.**
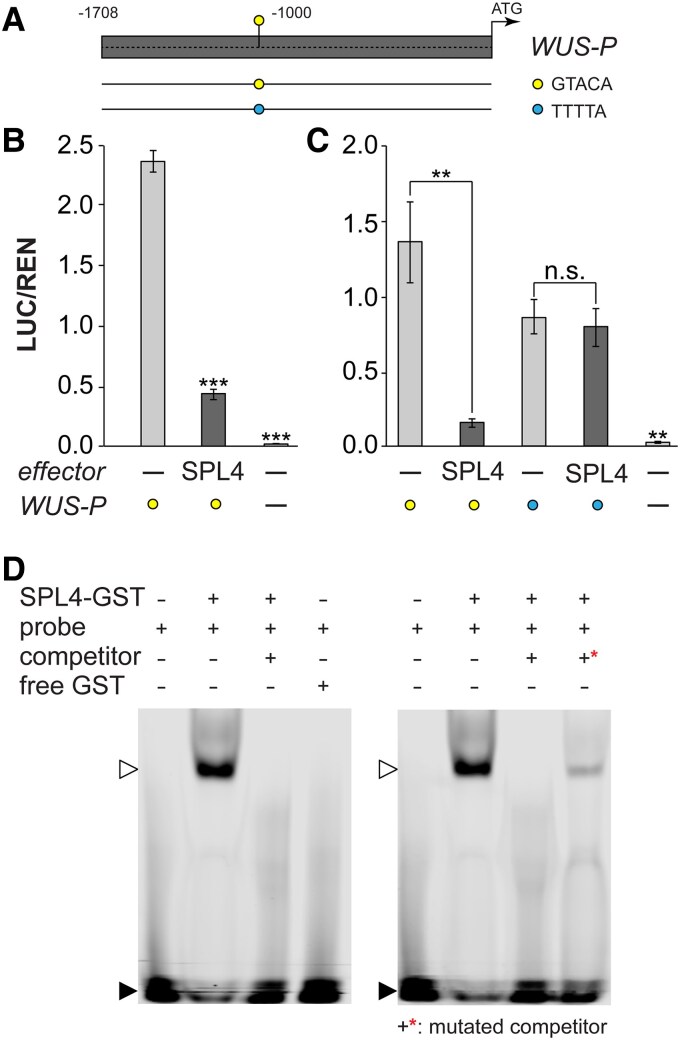
A negative feedback regulation between WUS and SPL4 in Arabidopsis. **A)** Overview of *WUS* 5′ regulatory region with putative *WUS^SPL^* site corresponding to the results shown in **B** to **D)**. Gray box indicates *5′ WUS* region with the native and mutated core *WUS^SPL^* site −1,067/−1,063 bp (indicated as circles). Sequence location and lengths used in **B**, **C)** indicated as lines below. **B**, **C)** Protoplast transactivation assay showing repression of the reporter (LUC) when coupled to the *5′WUS* region indicated in **A)**, **B)** relative to REN activity of the native *5′WUS*, and **C)** the comparison of the native and the mutated *WUS^SPL^* site, *n* = 3. **D)** EMSA for SPL4 binding to the *WUS^SPL^* site **A)**. Shifted band in the presence of SPL4 protein (open arrowhead) and nonshifted fraction (closed arrowhead). Please note the shift in the presence of the mutated competitor (+*), indicating specificity of binding. LUC, luciferase; REN, renilla. Error bars denote Sd; significance calculated based on 1-way ANOVA; **P < 0.01 and ***P < 0.001.

Since *WUS* and *SPL4* expression domains are largely overlapping (Yadav et al. 2011; Torti et al. 2012; Daum et al. 2014; Olas et al. 2019), we asked whether a feedback loop, involving direct regulation of *WUS* by SPL4, would explain the observed expression patterns. The SQUAMOSA PROMOTER BINDING PROTEIN (SBP) domain consists of highly conserved amino acids, which form the recognition motif for interaction with a GTAC core DNA sequence (Cardon et al. 1999; Birkenbihl et al. 2005). We identified a single putative *WUS^SPL4^* site −1,067-bp upstream of the *WUS* coding sequence (Fig. 6A). SPL4 significantly and specifically reduced reporter gene expression in an Arabidopsis protoplast-based transactivation assay, indicating that it can repress *WUS* in vivo (Fig. 6, B and C).

Confirming the result, EMSA illustrated specific binding of SPL4 to the investigated sequence, which was abolished when a mutated competitor sequence was used (Fig. 6D). This result demonstrates a likely transient, negative feedback regulation between SPL4 and WUS during floral transition, which is when *SPL4* is induced at the SAM (Schmid et al. 2003).

## Discussion

**Figure 7.**
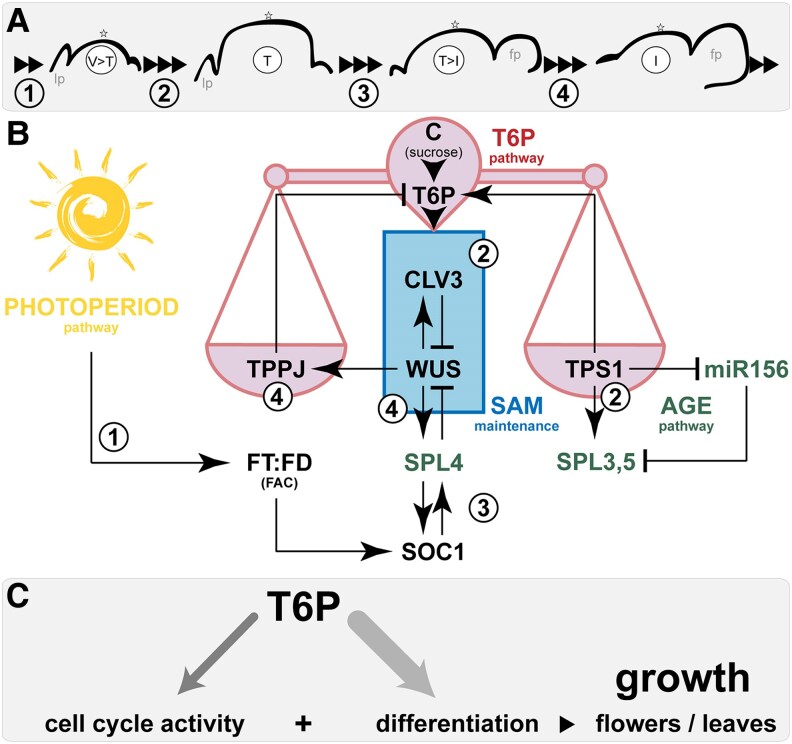
Dynamic regulations between sugar signaling, meristem maintenance, and the flowering network at the Arabidopsis SAM. **A)** Timing of SAM morphology throughout the floral transition. **B)** 1: Sugar **C)** signaling through the T6P pathway plays a central role in the induction of flowering in leaves via *FT* (downstream of the photoperiod pathway), but also at the SAM. 2: During vegetative development, SAM maintenance is controlled by WUS and CLV3 in a negative feedback loop. At floral transition, increased sucrose flux to the SAM and activity of the T6P pathway uncouples this regulation apart from its effect on the miR156/SPL module (age pathway). 3: This results in a spatial relocation of the *WUS* expression domain and doming and induces *SOC1* (floral integrator network). 4: WUS induces *SPL4* in the SAM center, which in turn represses *WUS* constituting a transient negative feedback loop. This allows the system to swing back, supported by WUS directly inducing *TPPJ* in the outer SAM layers. **C)** Impact of the T6P pathway on growth. leaf primordium (lp), flower primordium (fp), vegetative (V), transition (T), and inflorescence (I) SAM, florigen activation complex (FAC).

The initiation of reproductive growth marks a prominent change in plant morphology and requires large-scale reorganization of SAM architecture (Fig. 7A) and sink-source relationships of all flowering plants. Within cells, sucrose is an important basis for energy production and biomass accumulation. To prevent starvation, energy-demanding developmental transitions such as the floral transition are tightly coordinated with endogenous sucrose availability through intricate signaling systems (Fernie et al. 2020). It is therefore not surprising that all stages of plant development rely on proper energy signaling.

SAM maintenance is controlled by meristem maintenance regulators throughout plant development. We show that the central negative feedback loop between WUS and CLV3 uncouples during floral transition (Fig. 7, A and B), which associates with increased levels of T6P (Wahl et al. 2013). As a result, the *WUS* domain is no longer restricted to the center of the SAM but stretches into L1 demonstrating unreported OC dynamics (Fig. 1D). This possibly renders its function independent of cell-to-cell movement and is thus faster, speed essential for proper timing of SAM doming at floral transition. Supporting this idea, *WUS* when constitutively expressed from the *CLV3* promoter into the stem cell niche increases the SAM to a size even larger than that of a *clv3* mutant. This is due to a massive proliferation of meristematic cells (Brand et al. 2002). Notably, organ production is stalled in these lines (Brand et al. 2002), which explains why *WUS* can only be allowed to be expressed outside of OC boundaries during a short period of time. Thus, the doming process is eventually terminated to allow organ production to resume.

It makes sense to assume that WUS itself is part of the mechanism that initiates the doming process and eventually causes the system to swing back and terminate the process. Although the otherwise stable interaction is clearly disconnected during the doming phase, also CLV3 must still play a critical role. Its expression domain increases, likely as a consequence of the changes at the beginning of transition, and remains enlarged when *WUS* is already confined within the OC in a young, still domed inflorescence SAM (Fig. 1D; 12 DAG). CLV3 is a repressor of *WUS* and thus its increased expression later in the process might be necessary to limit *WUS* expression back to the OC. However, this does not exclude the possibility that WUS protein is still present in L1 beyond 12 DAG. It should be noted that CLV3 also indicates a stem-cell respecification by WUS (Reddy and Meyerowitz 2005). CLV3 function however does not seem to be essential for floral transition, as mutant plants, though delayed, eventually do bolt and form flowers (Fig. 4C; Supplementary Table S1). This is even the case in the strong *clv3-10* allele, which has an enormous and disorganized SAM in particular posttransition to flowering (Forner et al. 2015).

Floral transition and thus doming associates with increased T6P levels, which stay elevated in an inflorescence SAM. This affects the age pathway via miR156 and in part indecently through *SPL3-5* as we have previously demonstrated (Wahl et al. 2013). However, it is important to note that for technical reasons, these measurements resulted from whole SAM tissue and thus do not allow the interpretation of the dynamics between functionally distinct SAM zones and cell layers or even individual cells. Until the technical boundaries are lifted, these questions have to be addressed molecularly. Here, we found that WUS directly regulates *TPPJ* (Figs. 3 and 7B), and *TPPJ* is expressed in the outer layers of the PZ throughout development (Fig. 2, A and E; Supplementary Figs. S6 and S17). Most other *TPP* genes are also expressed in distinct SAM domains often in a stage-dependent manner (Supplementary Fig. S6), which might indicate that T6P accumulates in certain areas and reducing it in others might be important for SAM function. This also suggests that some areas are more responsive to the signal than others.

That T6P function in the outer SAM layers is important is supported by the earlier flowering and reduced SAM size phenotype of plants suppressing *TPPJ* in L1 only (Fig. 4, A and B; Supplementary Fig. S17, C and E, and Table S1). TPP dephosphorylates T6P to trehalose, thus reducing the abundance of the signal. The T6P pathway was previously suggested to have a positive effect on cell cycle regulators, as *tps1* embryos cease developing at torpedo stage of embryogenesis supposedly due to a lack of expression of important cell cycle regulators (Gómez et al. 2006). These were also demonstrated as differentially expressed at the transition stage (Klepikova et al. 2015). In line with this, the deregulation of *TPPJ* has an impact on SAM size (Fig. 2F and 4A; Supplementary Fig. S17) and seems important for cell proliferation and doming during the transition phase. The action of the T6P pathway however leads to a smaller SAM, e.g. when *TPS1* is overexpressed directly in the stem cell niche (Figs. 1, B and C) or when *TPPJ* is downregulated in L1 (Fig. 4A). Vice versa, downregulation of *TPS1* (Fig. 1, B and C) or misexpression of *TPPJ* in L1 results in a larger SAM (Supplementary Fig. S17). This is counterintuitive, as e.g. *CYCD3* misexpression, which induces cell cycle activity, results in significantly increased SAM size and accelerated development of all stages and vice versa a triple mutant in all 3 *CYCD3* loci leads to a reduced size (Boucheron et al. 2005; Dewitte et al. 2007). We can only suspect that cell proliferation and differentiation are simultaneously induced by the T6P pathway (Fig. 7C), leading to an increased cell output rate especially in transgenic plants with nonphysiological signaling activity.

*TPPJ* is activated by WUS in vivo (Figs. 4 and 7B). Cell nonautonomous WUS is known to both activate and repress target genes and, at low levels, such as at the rim of the PZ, was found to rather induce than suppress transcription (Perales et al. 2016). Similar to its effect on *CLV3* (Brand et al. 2002), WUS does not activate *TPPJ* outside of the outer layers, suggesting other factors repressing it in the other parts of the SAM. These factors are clearly active in a *clv3* mutant background, where the activation of *TPPJ* by WUS remains restricted to the outer layers (Fig. 2A).

miR156 and the targeted *SPL* transcripts, in particular *SPL3-5*, have emerged as targets of nutrient signals (Wahl et al. 2013; Yang et al. 2013; Yu et al. 2013; Olas et al. 2019; Ponnu et al. 2020; Zacharaki et al. 2022). This does explain the varying mutant phenotypes individual laboratories were reporting of, reflecting differing growth conditions, e.g. light, temperature, substrate, and irrigation regimes. We and others now irrefutably demonstrate the importance of SPL3-5 in the regulation of flowering through a CRISPR/*Cas9* approach (this study; Xie et al. 2020).

A study by Fouracre and Poethig (2019) discovered that a *wus-5* mutant had decreased miR156 levels, and while plants with reduced miR156 levels (quadruple *mir156a,c,mir157a,c*) had a smaller SAM, plants with increased miR156 levels (*35S:miR156a*) had a larger SAM. In line with this, we detected increased miR156 abundance in a *clv3* SAM; however, there was no effect on miR156 in the SAM of *wus-7* (Fig. 5, A and E), a weak allele with a mutation in the WUS DNA-binding domain resulting in reduced but still functional SAM tissue (Graf et al. 2010; Lin et al. 2016). In contrast, *wus-5* is a strong *wus* null allele with very little residual SAM tissue (McElver et al. 2001; Sonoda et al. 2007), which might explain the difference.

We previously identified the age pathway as a target of the T6P pathway at the SAM. Here, we believe both act in unison as a fail-safe to ensure reproductive success in the presence of sufficient carbohydrate resources to support the energy-demanding floral transition or in the absence of other inductive signals. The interaction occurs partially via miR156 and in part through other previously unknown direct effects on the expression of *SPL3-5* (Wahl et al. 2013). In this context, it is important to note that the interaction that controls timing and length of the doming phase involves SnRK1—a kinase at the center of energy management and a direct target of T6P signaling (Baena-González and Lunn 2020; Zacharaki et al. 2022). We further dissected the processes involved and demonstrate that SPL4, which is expressed in a domain overlapping with the OC, directly and negatively regulates *WUS* (Figs. 5, F to H, and 7B). At transition, negative feedback regulation between WUS and SPL4 is favored over WUS and CLV3 for a short period of time (Figs. 5 and 7B). This interaction is supported by a promoter study analyzing a series of deletion constructs of the 5′*WUS* sequence (Baurle and Laux 2005). Among them one that had the *WUS^SPL4^* site removed (Δ4, −941/−604 from transcription start site; −1,067/−730 upstream of coding sequence). Importantly, this deletion construct led to a stronger activation while others rather led to a reduction of the GUS reporter, especially when affecting the sequences close to the transcription start site. This indicates the loss of regulatory sequences that are targeted by repressors of *WUS* such as SPL4 (Baurle and Laux 2005).

However, similar to *CLV3* and *TPPJ*, *SPL4* cannot be activated outside of its central expression domain (Supplementary Fig. S21). Importantly, *SPL4* is induced much earlier in a *clv3* than in a wild-type SAM (Supplementary Fig. S21), further supporting a likely transient, negative feedback regulation between WUS and SPL4. Surprisingly, if *SPL4* is considered as an early flowering marker, its earlier induction in the center of a *clv3* SAM also accounts for an early onset of floral transition in *clv3*. Taken together with the otherwise delayed flowering phenotype (Fig. 4C; Supplementary Table S1), this indicates an extension of the transition phase in the mutant and would support a role of CLV3 in restricting the doming process. In this scenario, *TPPJ* repression in L1 might in fact lead to a shortening of the transition phase and explain the overall reduced SAM size and early flowering phenotype (Fig. 4, A and B; Supplementary Fig. S17 and Table S1). If this is indeed the case remains to be determined in the future.

In summary, we proposed a simple chain of events (Fig. 7, A to C). At the beginning of the transition phase, *FT* is induced in leaves as controlled by exogenous and endogenous factors, e.g. the photoperiod pathway, but also depending on the activity of the T6P pathway. FT mobility to and its presence at the SAM induce floral transition. This starts with the initiation of spatial changes of *WUS* expression, which result in increased cell cycle activity, cell proliferation, and expansion of the SAM, during which organ production (leaves) is stalled. These events are high energy demanding and require increased sucrose unloading, which results in increased T6P and modulation of the flowering network, likely reinforced via a positive feedback loop between the age pathway and SUPPRESSOR OF OVEREXPRESSION OF CONSTANS 1 (SOC1). Toward the end of the transition stage, *WUS* expression is confined back to its original domain to allow regular cell cycle activity and proliferation before organ production resumes (now flowers). This is achieved by increased repression through *CLV3* and *SPL4*. In this scenario, the TPS1/TPPJ hub effectively works to balance the system, which allows swinging out of (homeostatic condition 1 = vegetative SAM) and back into a stable WUS/CLV feedback loop (homeostatic condition 2 = inflorescence SAM) through feedback regulations “external” of the central core. In summary, the dynamic feedback regulations we discovered ensure speeding up of cell cycle activity and metabolism at floral transition and provide the basis and energy necessary to expand the SAM. This process is critical for floral induction.

Apart from the regulation of flowering time (Wahl et al. 2013; Olas et al. 2019; Gramma et al. 2024), it is remarkable that the T6P pathway as controlled by WUS also influences the reorganization of the SAM during floral transition. Important next steps will be to understand the role of a potential moonlighting function of TPP proteins (Claeys et al. 2019) and how SnRK1- and TOR-dependent signaling (Pfeiffer et al. 2016; Caldana et al. 2019; Baena-González and Lunn 2020) fit into the emerging picture of the mechanisms regulating the timing of the processes at the SAM during floral transition. Lastly, given the ubiquitous nature of carbohydrate signaling and the large-scale change in sink–source relationships within plants (Fernie et al. 2020), it will be interesting to determine if this regulatory mechanism is widely present in the plant kingdom, which would make it an ideal breeding target for improved plant architecture and crop yield.

## Materials and methods

### Plant material and growth conditions

Arabidopsis plants are of the Columbia accession (Col-0), except for *wus-7*, which is in the L*er* background (Graf et al. 2010). *clv3-7* (Wisman et al. 1998), *clv3-10* (Forner et al. 2015), *35S:amiRTPS1*, *CLV3:TPS1* (Wahl et al. 2013 ), and *TCSn:GFP* (Zurcher et al. 2013) were described previously. The *tppj* insertion mutant (At5g65140, GABI_215C08) was obtained from the Arabidopsis Biological Resource stock Center. Growth chamber settings were 22 °C in LD (16 h light/8 h dark), SD (8 h dark/16 h light), light intensity ca. 160 *μ*mol/m^−2^s^−1^, and relative humidity 60% to 65%. Synchronized induction of flowering was performed as described (Schmid et al. 2003).

### Plant phenotyping

Flowering time of on average of 20 plants per genotype was scored as days to flowering, when shoots were 0.5 cm (bolting), and by the total leaf number, i.e. the sum of rosette leaf and cauline leaf numbers (Supplementary Table S1). Meristem size was measured as the area between 2 organ primordia under the meristem summit of a longitudinal middle section through at least 10 apices of individual plants using the Fiji software version 2.0.0-rc-69/1.52 (Schindelin et al. 2012).

### Generation of transgenic lines

Plants were transformed (Clough and Bent 1998), confirmed by PCR, and independent, single-insertion, homozygous T3 plants were used for all studies. Oligonucleotides for cloning and genotyping are given in Supplementary Tables S2 and S3. For *35S:TPPJ* and *35S:TPS1*, coding sequences of *TPPJ* (At5g65140) and *TPS1* (At1g78580) were cloned via the Gateway entry vector *pJLBlue* reverse (Mathieu et al. 2007) into a *pGREEN-II*-based destination vector. Artificial microRNAs targeting *TPPJ* (*ML1:amiRTPPJ* V1, V2; *35S:amiRTPPJ* V1, V2) were designed with the MicroRNA Designer (http://wmd3.weigelworld.org/cgi-bin/webapp.cgi) (Schwab et al. 2006) and cloned via *pJLBlue* reverse (Mathieu et al. 2007) into a *pGREEN-II*-based destination vector with the *ML1* or *35S* promoter. *spl3, spl4, spl5* knockout lines were generated with the CRISPR/*Cas9* technology (Wang et al. 2015; Ruf et al. 2019). The NGG PAM recognition sites were defined using ATUM (https://www.atum.bio) (Supplementary Fig. S22). The *pJF1033* vector, containing a 2 single guided RNA scaffold, was used as a template. *Bsa*I products were cloned into the *pJF1031* binary vector (Ruf et al. 2019). Plants with homozygous deletions were back-crossed to Col-0. Homozygous, *Cas9*-free plants were used to generate all higher-order mutants.

### RT-qPCR

Total RNA was isolated by a modified phenol/chloroform method using a modified TRIzol reagent, followed by sodium acetate precipitation. Genomic DNA was removed with RNase-free DNase I (Ambion/Thermo Fisher Scientific, Waltham, Massachusetts, United States), and cDNA synthesis was carried out using a SuperScript IV Reverse Transcriptase Kit (Thermo Fisher Scientific, Waltham, Massachusetts, United States). Mature miR156 stem-loop primers (Supplementary Table S4) were added to the cDNA synthesis reaction (1:1 with oligo dT(18) primer) (Varkonyi-Gasic et al. 2007).

Reverse transcription qPCR (RT-qPCR) for expression analyses and ChIP-qPCRs was performed with the ABI Prism 7900 HT fast real-time PCR system (Applied Biosystems/Life Technologies, Darmstadt, Germany) using a Power SYBR Green-PCR Master Mix (Applied Biosystems/Life Technologies, Darmstadt, Germany). Oligos are listed in Supplementary Table S4 (RT-qPCR) and Supplementary Table S5 (ChIP-qPCR). The SDS 2.4 software (Applied Biosystems/Life Technologies, Darmstadt, Germany) was used for analysis (Czechowski et al. 2004). cDNA quality was determined and expression values were calculated (Wahl et al. 2013).

### RNA in situ hybridization

Probe cloning (Supplementary Table S6), synthesis, wax embedding, sectioning, RNA in situ hybridization, and imaging were performed as described (Wahl et al. 2013; Gramma and Wahl 2023).

### ChIP-qPCR

For ChIP-qPCR, 100 apices or 1.5-g leaves per replicate (Col-0, *clv3-7*, and *clv3-10*) were fixed in 1% (*v*/*v*) formaldehyde buffer (10 mm sodium phosphate buffer, pH7; 50 mm NaCl; 100 mm sucrose) under vacuum. ChIP was performed (Kaufmann et al. 2010) with modifications: antibody incubation (anti-WUS; AS11 1759; Agrisera) was extended o/n at 4 °C, Agarose beads (Protein A-Agarose; sc-2001; Santa Cruz Biotechnology) to 6 h at 4 °C. Immunoprecipitated DNA was analyzed by qPCR. Ct values of *TPPJ* and *SPL4* promoter regions were normalized to the Ct value of a *UBQ10* promoter region. The % of enrichment was calculated as relative to expression of the input of the individual regions. ChIP on apices of Col-0, *clv3-7*, and *clv3-10* without antibody and ChIP on Col-0, *clv3–7*, and *clv3-10* leaves were controls (Supplementary Fig. S10). Note that the WUS antibody detects confirmed WUS target sites (Supplementary Fig. S12) (Leibfried et al. 2005; Yadav et al. 2011), and no amplification was observed in the negative controls (Supplementary Figs. S10 and S23). Oligonucleotides are listed in Supplementary Table S5.

### Transactivation assay

For the effector line, the *WUS* (At2g17950) and *SPL4* (At1g53160) CDSs were cloned via the Gateway entry vector *pJLBlue* reverse (Mathieu et al. 2007) into the *pMDC32* Gateway vector (pMML058) and the *pGW5* (pKK77). For the reporter constructs, designated *5′TPPJ* regions were *Kpn*I/*Acy*I cloned into the Gateway *pMDC162* vector (GUS reporter). The *5*′*SPL4* and *5′WUS* regions were *Kpn*I/*Spe*I cloned into *pGREEN800II-LUC* (firefly luciferase reporter; Hellens et al. 2005) (Supplementary Table S2). Protoplasts were isolated from 4-wk-old plants, transfected (Wu et al. 2009), and incubated (20 h, 22 °C, 100 *µ*mol/m^−2^s^−1^). Luciferase activity of the *5′TPPJ*, *5*′*SPL4*, and *5′WUS* assays was measured with a luciferase and dual-luciferase reporter assay system, respectively (Promega, Madison, Wisconsin, United States). In the *5′TPPJ* assays, the *35Somega:LUC-NOS* vector was used as a control for background readout. GUS activity was determined as described (Yoo et al. 2007).

### EMSA

The *WUS* CDS without STOP codon was cloned via the Gateway entry vector *pDONR207* (pMML059) into a Gateway destination vector (*pDEST24*). *35S:WUS-GST* (pMML063) was transformed into *Escherichia coli* Rosetta plysS cells and protein production induced with 1 mm isopropyl β-D-1-thiogalactopyranoside at 30 °C o/n. Cell lysis was performed with 1× sonication (5 s, 20% power, 4 cycles, Sonopuls Hd 2070 Sonicator, Bandelin, Berlin, Germany) in a freshly prepared buffer (20 mm Na-phosphate buffer, pH7.4; 0.5MNaCl; 1 mm phenylmethylsulfonyl fluoride; 1 mm ethylenediaminetetraacetic acid; 1 tablet cOmplete Protease Inhibitor Cocktail [Merck, Darmstadt, Germany] per 10-ml buffer). Two-microgram protein (crude extract, Pierce BCA Protein Assay Kit, Thermo Fisher Scientific, Waltham, Massachusetts, United States) was used for the EMSA; 50-bp, double-stranded, 5′-IRDey-682-labeled probes, spanning the putative *TPPJ^WUS^* and *WUS^SPL4^* sites, are listed in Supplementary Table S7 (5′*TPPJ*) and Supplementary Table S8 (5′*WUS*). Binding reactions were carried out with the Odyssey EMSA Kit with a competitor and/or mutated competitor to probe ratio of 1:200 and visualized with the Oddysey Infrafed Imaging System (Li-Cor, Lincoln, Nebraska, United States).

### Confocal microscopy

SAMs of a *TCSn:GFP* cytokinin reporter line (Zurcher et al. 2013) were imaged with a Leica TCS SP8 confocal laser scanning system (Leica, Wetzlar, Germany) equipped with a M6000B-CS microscopy stage, an Argon laser (65 mV), and a 40× water immersion HCX APO objective as follows: laser output power 20%; GFP excitation (green, wavelength 488 nm), emission detection channel 3 (495 to 520 nm), and gain PMT 800 V; plastid auto-fluorescence (blue), emission detection channel 4 (700 to 800 nm), and gain PMT ∼500 V; scan speed 600 Hz in xyz bidirectional scanning mode with a z-stack distance of approx. 10 *µ*m. Offset = 0; pixel dimension: 1,024 × 1,024. Middle sections of representative SAMs were extracted from z-stacks using the Fiji software version 2.0.0-rc-69/1.52 (Schindelin et al. 2012).

### Statistical consideration

Statistical significance was analyzed by a 1-way ANOVA with Tukey’ post hoc honestly significant difference based on Tukey–Kramer correction (*P* < 0.05) or a 2-tailed Student's *t*-test.

### Accession numbers

CLV3 (At2g27250), ML1 (At4g21750), SPL3 (At2g33810), SPL4 (At1g53160), SPL5 (At3g15270), TPPJ (At5g65140), TPS1 (At1g78580), and WUS (At2g17950).

## Supplementary Material

kiaf300_Supplementary_Data

## Data Availability

All data supporting the findings are contained in this manuscript. Additional supplementary data may be found online in the Supplementary data section at the end of the article. Materials supporting the findings of this study are available from the corresponding author upon reasonable request.

## Supplementary data

The following materials are available in the online version of this article.

**Supplementary Figure S1.**  *TPS1* expression at the shoot apical meristem of *35S:amiRTPS1* plants.

**Supplementary Figure S2.**  *TPS1* expression at the shoot apical meristem of *35S:TPS1* and *CLV3:TPS1* plants.

**Supplementary Figure S3.** Metrics at the shoot apical meristem before and at the floral transition.

**Supplementary Figure S4.** Shoot apical meristem area in short- to long-day-shifted plants and *WUS* expression in short days.

**Supplementary Figure S5.** Analysis of the cytokinin signaling reporter TCSn:GFP.

**Supplementary Figure S6.** Expression of Arabidopsis *TPP* genes at the shoot apical meristem detected by RNA in situ hybridization.

**Supplementary Figure S7.** AtGenExpress expression profiles of the Arabidopsis *TPP* genes.

**Supplementary Figure S8.** Expression of meristem maintenance genes and *TPPJ* in the *clv3-7* and *clv3-10* mutant shoot apical meristem.

**Supplementary Figure S9.**  *TPPJ* Expression in *WUS* transgenic lines.

**Supplementary Figure S10.** ChIP-qPCR control experiments for Fig. 3, C to E.

**Supplementary Figure S11.** ChIP-qPCR on known WUS targets using a specific WUS antibody.

**Supplementary Figure S12.** ChIP-qPCR on G-box like WUS binding sites.

**Supplementary Figure S13.** Electrophoretic mobility shift assay (EMSA) control experiments.

**Supplementary Figure S14.** Expression of *TPP* genes in *tppj* mutant plants.

**Supplementary Figure S15.** Expression of *TPP* genes in *35S:amiRTPPJ* plants.

**Supplementary Figure S16.** Earlier flowering of *35S:amiRTPPJ* and *tppj* lines.

**Supplementary Figure S17.** Importance of TPPJ in the outer meristem layer.

**Supplementary Figure S18.** Wild-type like flowering phenotype when *ML1:amiRTPPJ* is introgressed into *clv3-10*.

**Supplementary Figure S19.**  *WUS* and *CLV3* expression in apices of trehalose lines.

**Supplementary Figure S20.** Expression of *SPL15* at the shoot apical meristem.

**Supplementary Figure S21.**  *SPL4* expression in *clv3* apices.

**Supplementary Figure S22.**  *SPL3*, *SPL4*, and *SPL5* mutant lines.

**Supplementary Figure S23.** Potential *SPL^WUS^* sites in the 5′ intergenic regions of *SPL3*, *SPL4*, *SPL5*, *SPL9*, and *SPL15*.

**Supplementary Figure S24.** ChIP-qPCR control experiments.

**Supplementary Figure S25.**  *WUS* and *CLV3* expression in apices of short day-grown SPL double and triple CRISPR/*Cas9* deletion mutants.

**Supplementary Table S1.** Flowering times of Col-0, mutants and transgenic plants.

**Supplementary Table S2.** Oligonucleotides for constructs used for the generation of transgenic lines and for transactivation assays.

**Supplementary Table S3.** Oligonucleotides used for genotyping.

**Supplementary Table S4.** Oligonucleotides used for RT-qPCR.

**Supplementary Table S5.** Oligonucleotides used for ChIP-qPCR.

**Supplementary Table S6.** Oligonucleotides used to prepare constructs for probe synthesis.

**Supplementary Table S7.** EMSA oligonucleotides (probes) for *5′TPPJ* At5g65140.

**Supplementary Table S8.** EMSA oligonucleotides (probes) for 5*′WUS* At2g17950.

## Dive Curated Terms

The following phenotypic, genotypic, and functional terms are of significance to the work described in this paper:

CLV3 Gramene: AT2G27250
CLV3 Araport: AT2G27250

SPL Gramene: AT4G27330
SPL Araport: AT4G27330

SPL9 Gramene: At2g42200
SPL9 Araport: At2g42200

SPL4 Gramene: AT1G53160
SPL4 Araport: AT1G53160

UBQ10 Gramene: AT4G05320
UBQ10 Araport: AT4G05320

SPL3 Gramene: AT2G33810
SPL3 Araport: AT2G33810

SPL5 Gramene: AT3G15270
SPL5 Araport: AT3G15270

WUS Gramene: AT2G17950
WUS Araport: AT2G17950

SPL15 Gramene: AT3G57920
SPL15 Araport: AT3G57920

EMSA Gramene: electrophoretic mobility shift assay
EMSA Araport: electrophoretic mobility shift assay
