# Supplementary material for: The trehalose 6-phosphate pathway coordinates dynamic changes at the shoot apical meristem in Arabidopsis thaliana
Source: Plant Physiol. 2025 Jul 10;199(1):kiaf300. doi: 10.1093/plphys/kiaf300 (PMC12415856; doi:10.1093/plphys/kiaf300)
Supplement: kiaf300_Supplementary_Data [file kiaf300_supplementary_data.zip › Supplementary Data.pdf]

Supporting Information

The T6P pathway coordinates Dynamic Changes at the Shoot Apical Meristem *in Arabidopsis thaliana*

Magdalena Musialak-Lange[1], Katharina Fiddeke[1,#], Annika Franke[1,#], Friedrich Kragler[1], Christin Abel[1], and Vanessa Wahl[1,2*]

Affiliations: [1]Max Planck Institute of Molecular Plant Physiology, Department of Metabolic Networks, Am Mühlenberg 1, Potsdam, 14476, Germany. [2]Present address: The James Hutton Institute, Errol Road, Dundee, DD2 5DA, United Kingdom.

[#]K.F. and A.F. contributed equally

[*]**Correspondence**: Vanessa Wahl

Email: vanessa.wahl@hutton.ac.uk

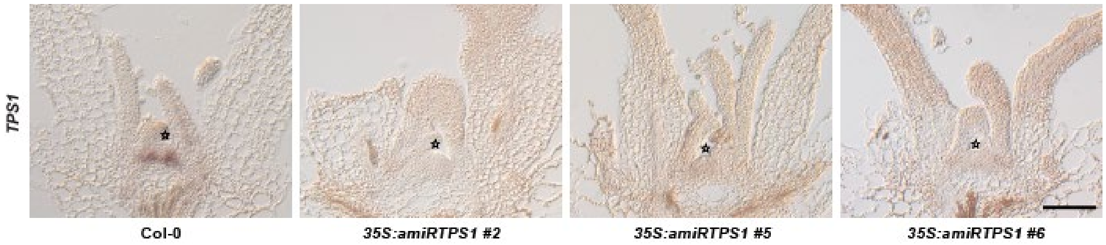

Supplementary Figure S1. *TPS1* expression at the shoot apical meristem of *35S:amiRTPS1* plants. Downregulation of *TPS1* by overexpressing an artificial microRNA targeting *TPS1* (*35S:amiRTPS1*) in plants of three independent lines (#2, #5 and #6; (Wahl *et al*, 2013)) as analyzed by RNA *in situ* hybridization using a specific probe against *TPS1*. Pictures depict representative longitudinal middle sections through vegetative apices (8 days after germination in long days) of Col-0 and *35S:amiRTPS1*, respectively. Line #6 was used for the experiments in this study. Star indicates shoot apical meristem summit. Scale bar is 50 µm**.**

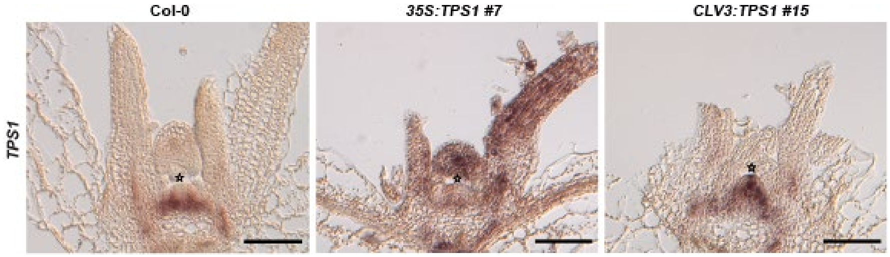

Supplementary Figure S2. *TPS1* expression at the shoot apical meristem of *35S:TPS1* and *CLV3:TPS1* plants. In comparison to expression in Col-0 *TPS1* was generally found upregulated in plants overexpressing *TPS1* (*35S:TPS1*, #7) and the central zone when expressing it under the control of the *CLAVATA3* (*CLV3*) promoter (*CLV3:TPS1*, #15) as analyzed by RNA *in situ* hybridization using a specific probe against *TPS1*. Pictures depict representative longitudinal middle sections through vegetative apices (8 days after germination in long days) of Col-0, *35S:TPS1*, and *CLV3:TPS1*, respectively. Star indicates shoot apical meristem summit. Scale bars are 50 µm.

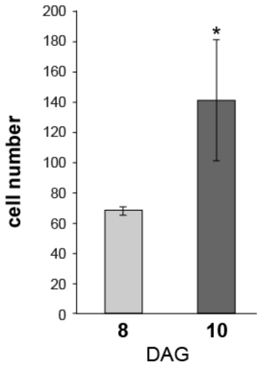

Supplementary Figure S3. Metrics at the shoot apical meristem (SAM) before and at the floral transition. Cell numbers as recorded at 8 (vegetative SAM) and 10 days (transition SAM) after germination (DAG) from single middle sections through apices of long day grown plants from at least 10 plants per time point. Significance calculated based on a one-way Anova; *P<0.05.

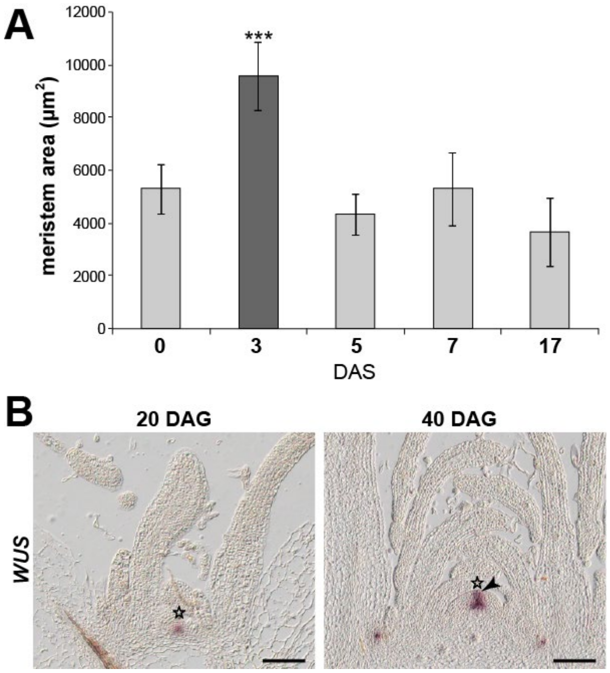

Supplementary Figure S4. Shoot apical meristem (SAM) area in short- to long-day-shifted plants and *WUS* expression in short days. (**A**) Vegetative and inflorescence SAM areas (light grey), and transition SAM area (dark grey) of wild-type plants grown in short day for 30 days and transferred to long day for 3, 5, 7 and 17 days (days after shift - DAS). (**B**) RNA *in situ* hybridization using a *WUS* specific probe on apices of short-day-grown plants at 20 and 40 days after germination (DAG). Arrow head indicates *WUS* expression in outer SAM layer. Error bars denote s.d.; ****P*<0.001 in relation to 0 days after transfer to long days (Student's *t*-test). Scale bars are 50µm. Stars depict SAM summit.

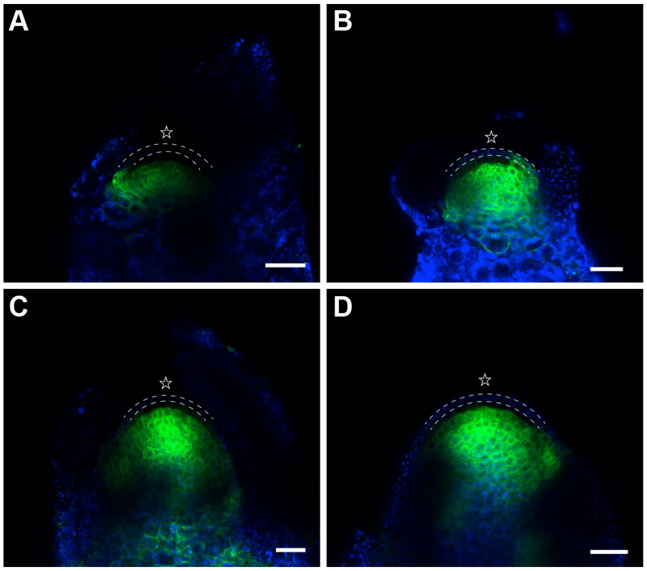

Supplementary Figure S5. Analysis of the cytokinin signaling reporter TCSn:GFP. Representative confocal laser scanning microscope images of shoot apical meristems of (**A**) 6, (**B**) 8, (**C**) 10, and (**D**) 12 days after germination of TCSn:GFP cytokinin reporter line (green (Zurcher *et al*, 2013); blue – autofluorescence of chloroplasts). Please note the absence of cytokinin signal in the L1 (between dashed lines) and L2 (cell layers below) in the meristem proper. Star indicates shoot apical meristem summit, scale bars are 25μm.

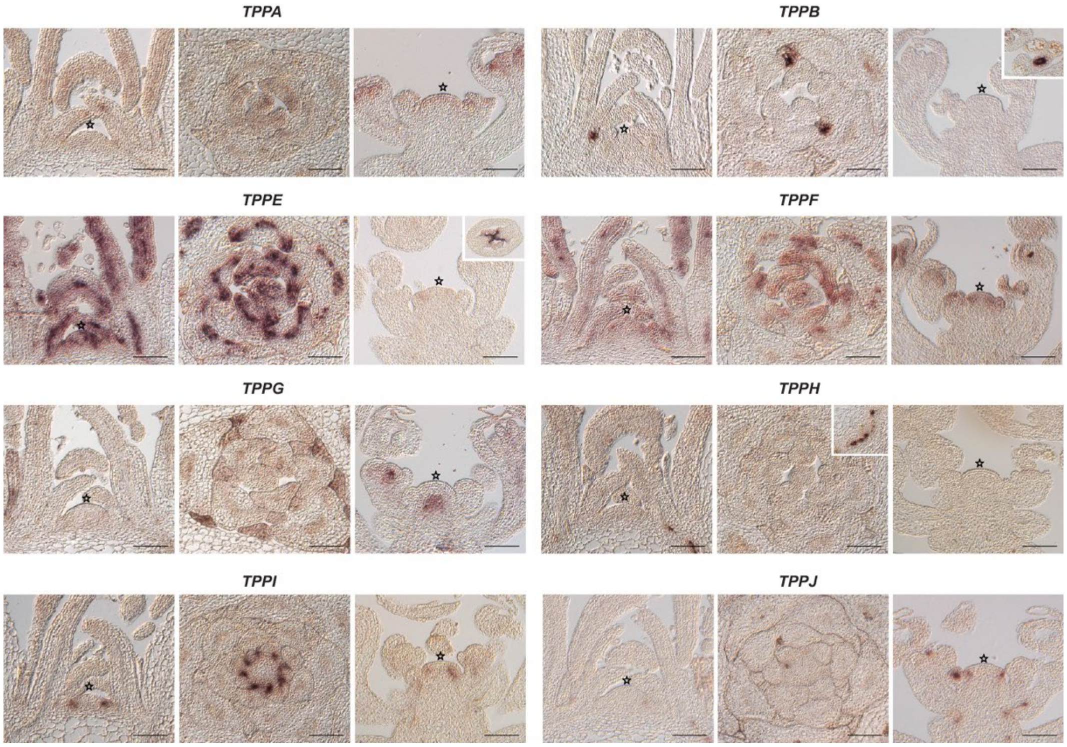

Supplementary Figure S6. Expression of Arabidopsis *TPP* genes at the shoot apical meristem detected by RNA *in situ* hybridization. The individual panels contain: a longitudinal middle section and a cross section through a vegetative Col-0 apex and a longitudinal middle section through an inflorescence apex. Representative pictures of *TPPC* and *TPPD* were omitted from this figure, since their specific probes gave no signal, indicating that transcripts of *TPPC* and *TPPD* were below detection limit in the tissues and/or stages analyzed. Star indicates shoot apical meristem summit. Scale bars are 100 μm.

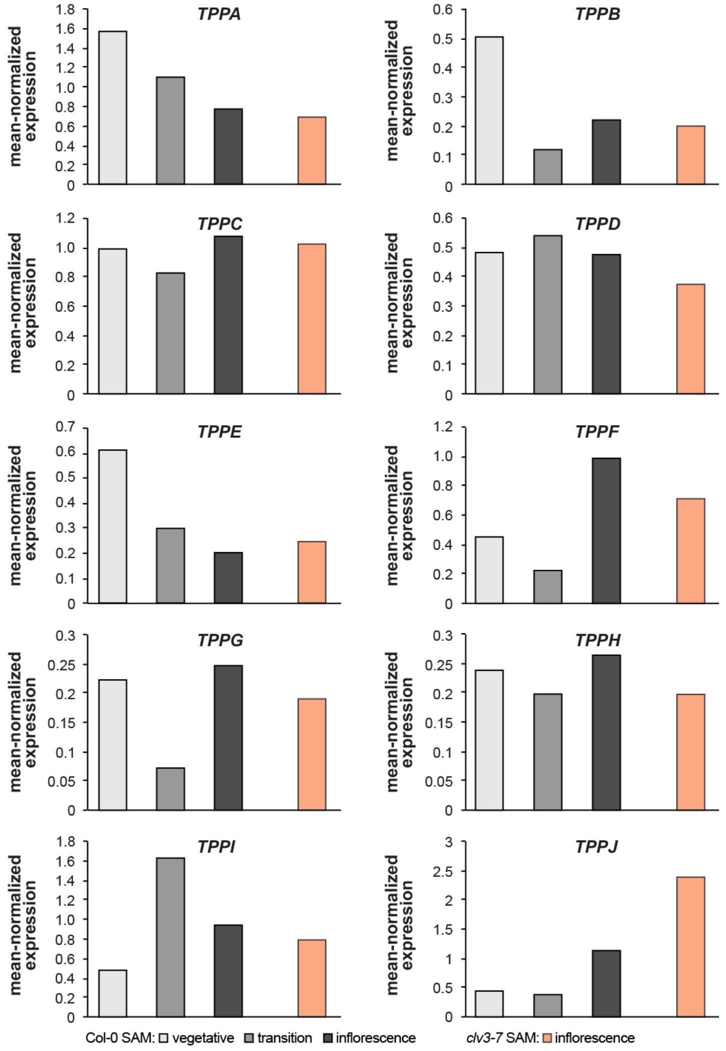

Supplementary Figure S7. AtGenExpress expression profiles of the Arabidopsis *TPP* genes. Values for Col-0 vegetative, transition and inflorescence apices (grey scale) and *clv3-7* inflorescence apices (orange) extracted from data deposited for (Schmid *et al*, 2005). All data points were mean-normalized.

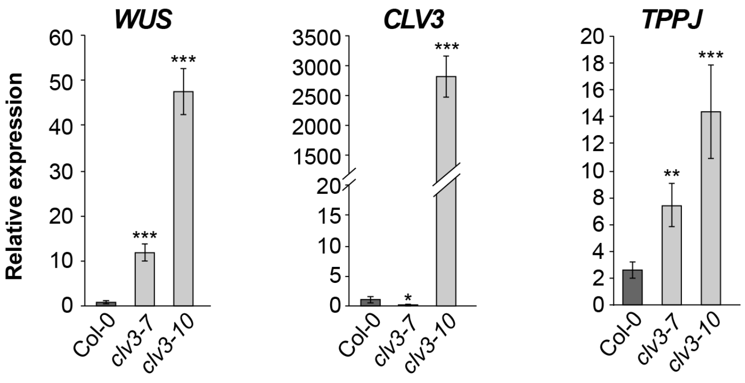

Supplementary Figure S8. Expression of meristem maintenance genes and *TPPJ* in the *clv3-7* and *clv3-10* mutant shoot apical meristem (SAM). RT-qPCR results of *TPPJ*, *WUS* and *CLV3* expression in short day-grown *clv3-7* and *clv3-10* SAMs, harvested 40 days after germination. Please note that the graph for *TPPJ* is a biological repetition of the graph in Figure 2C. Please also note that *clv3-10* is a TALEN generated mutant with a five nucleotide deletion in the last exon, which renders the transcript inactive to produce a functional protein (Forner *et al*, 2015). This is the cause for increased expression of *CLV3* downstream of increased *WUS* in the *clv3-10* mutant background. Error bars denote s.d.; **P*<0.05, ***P*<0.01, ****P*<0.001 (one-way ANOVA).

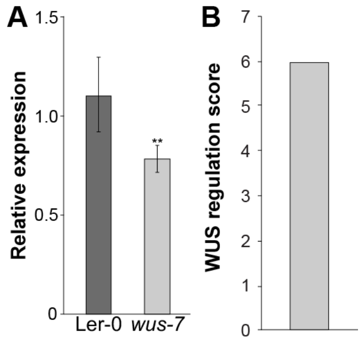

Supplementary Figure S9. *TPPJ* Expression in *WUS* transgenic lines. (**A**) RT-qPCR results of *TPPJ* expression in short day-grown *wus-7* as compared to wild-type Ler-0 shoot apical meristems. Error bars denote s.d.; **P<0.01, (one-way ANOVA). (**B**) *TPPJ* expression is increased upon WUS induction as shown by a WUS regulation score (WRS). According to Busch and colleagues, the WRS quantifies gene expression in response to a modulation in WUS activity normalized to the average response of all genes. Values extracted form a data set, deposited as a supplemental material of a publication by (Busch *et al*, 2010).

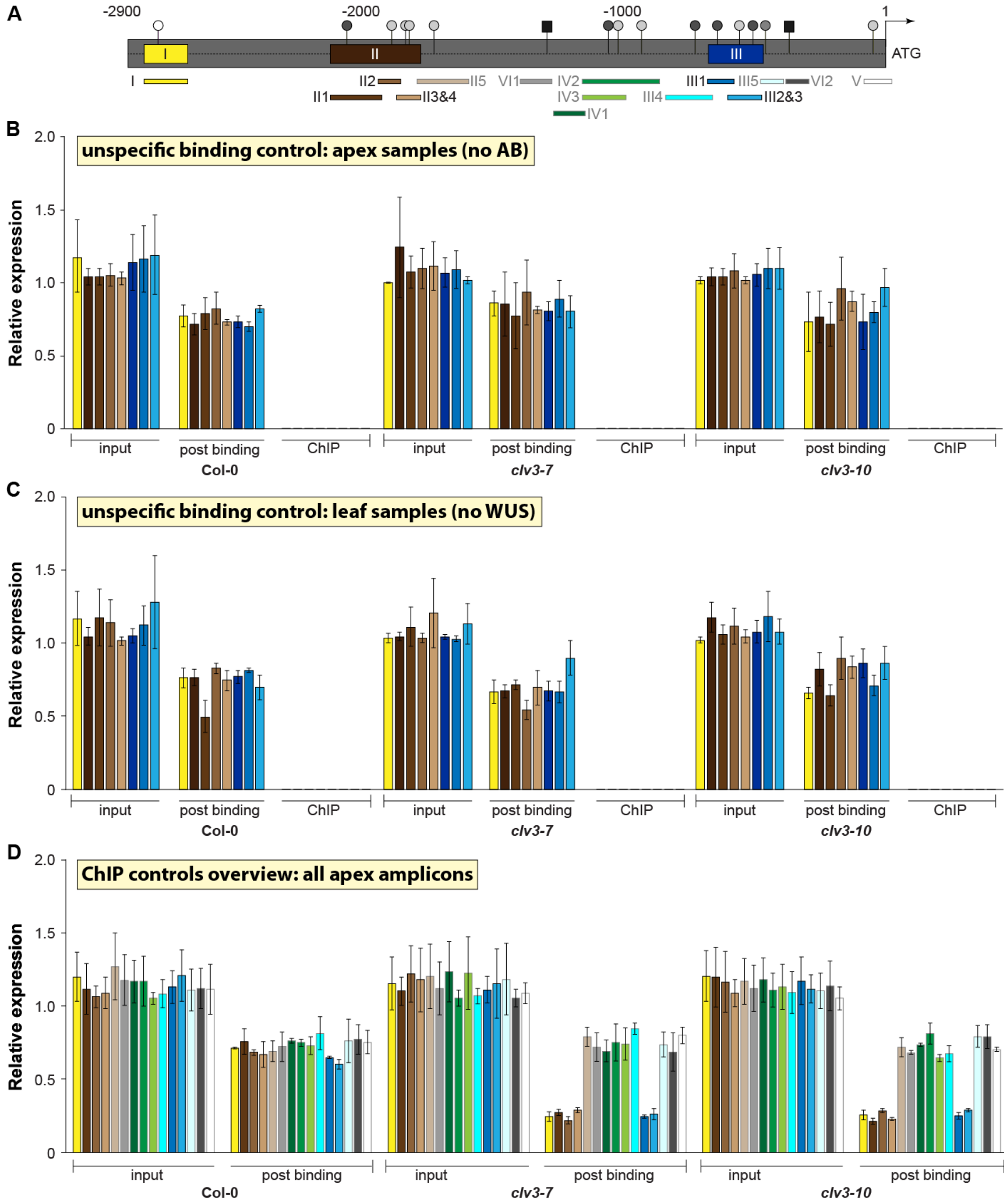

Supplementary Figure S10. ChIP-PCR control experiments for Figure 3c-e. (**A**) Overview of *5'TPPJ* intergenic sequence with all investigated putative $TPPJ^{WUS}$ sites (grey circles, black boxes), position of ChIP-PCR amplicons corresponding to the results shown in (B-D). Black framed boxes marked with I, II (1-4), and III (1-3) indicate *5' TPPJ* regions directly bound by WUS – I: -2795 – -2789 bp, II: -2073 – -1830 bp, and III: -652 – -564 bp, as presented in the Figure 3. Grey framed boxes (II5, III3-4, IV1-3, V1, VI1-2) represent *5'TPPJ* regions not directly bound by WUS. (**B-D**) Relative expression of investigated regions (I-III) containing $TPPJ^{WUS}$ elements (I, II1-4, III1-2) in Col-0, *clv3-7* and *clv3-10* (**B**) shoot apex samples without WUS antibody, and (**C**) leaf samples without WUS were used as specificity controls. Please note the amplification in the input and post-binding fractions (PB) and the absence of amplification in the ChIP samples indicating specificity of the antibody used. (**D**) Control overview of all investigated putative $TPPJ^{WUS}$ sites in input and post binding fraction samples. Error bars denote s.d.

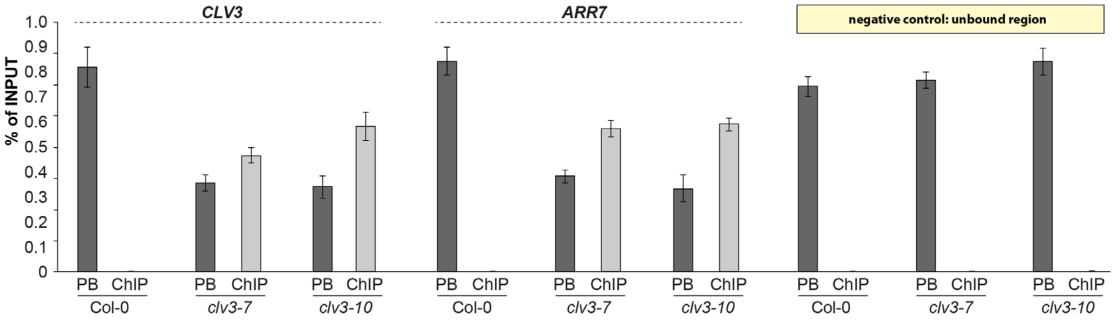

Supplementary Figure S11. ChIP-PCR on known WUS targets using a specific WUS antibody. Relative to the input enrichment of known *CLV3*^{WUS} (Yadav *et al*, 2011) and *ARABIDOPSIS RESPONSE REGULATOR 7* (*ARR7*^{WUS}) (Leibfried *et al*, 2005) in *clv3-7* and *clv3-10* as measured by ChIP-PCR. As a negative control a *5'TPPJ* region was used that did not show any WUS binding (unbound region). Error bars denote s.d.

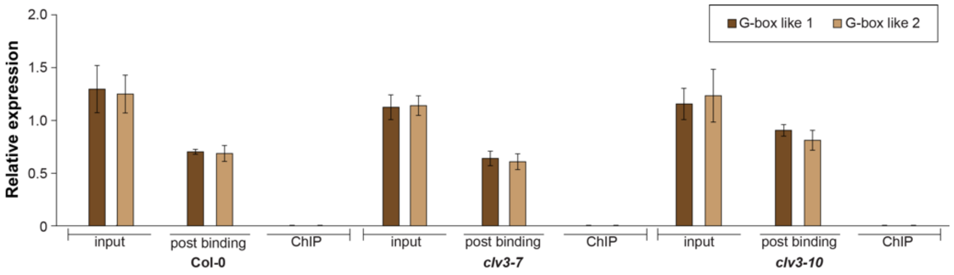

Supplementary Figure S12. ChIP-PCR on G-box like WUS binding sites. Relative expression of two G-box like WUS binding regions (CACGTG) (Sloan *et al.*, 2020) present in the *5'TPPJ* intergenic region in input, post binding and ChIP samples of Col-0, *clv3-7* and *clv3-10*. G-box like 1 and G-box like 2 correspond to the regions VI1 and VI2 of Fig. S11A. Please note the amplification in the input and post-binding fractions and the absence of amplification in the ChIP samples. Error bars denote s.d.

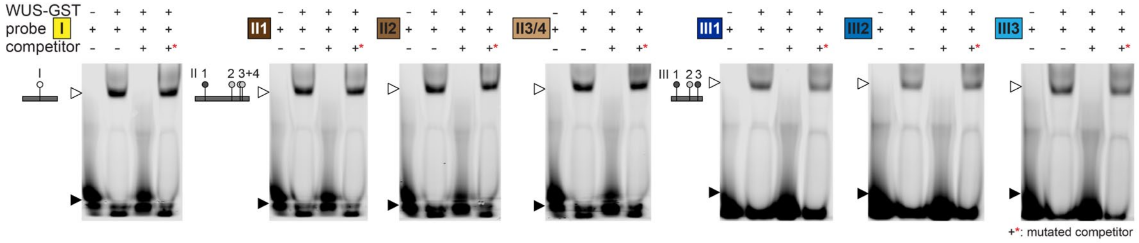

Supplementary Figure S13. Electrophoretic mobility shift assay (EMSA) control experiments. EMSA for WUS binding to the indicated regions (I-III) in Fig. 3A as a control for Fig. 3F. Shifted band in the presence of WUS protein (open arrow head), non-shifted fraction (closed arrow head). Please note the shifted band in the presence of the mutated competitor (+*, Table S8) indicating specificity of the binding.

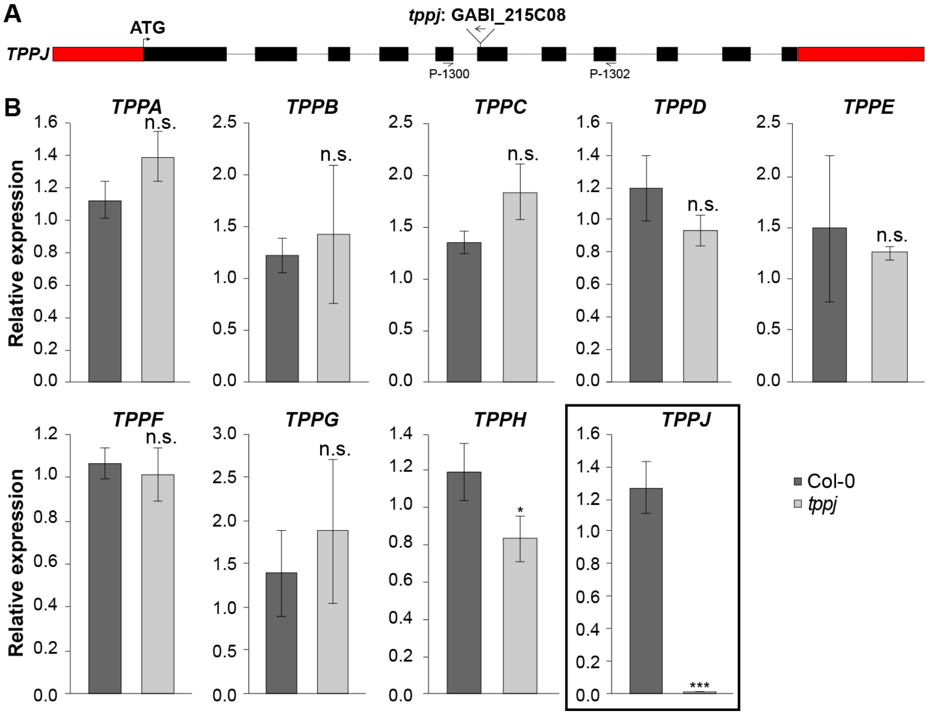

Supplementary Figure S14. Expression of *TPP* genes in *tppj* mutant plants. (**A**) Schematic illustration of the T-DNA insertion (GABI_215C08) in the 6th exon of *TPPJ* including positions of oligos used for genotyping. (**B**) Expression of *TPPs* in long day-grown Col-0 and *tppj* rosettes, harvested without roots at 10 days after germination as measured by RT-qPCR. In this experiment *TPPI* was not detectable. Error bars denote s.d.; *P<0.01; ***P<0.001 (one-way ANOVA).

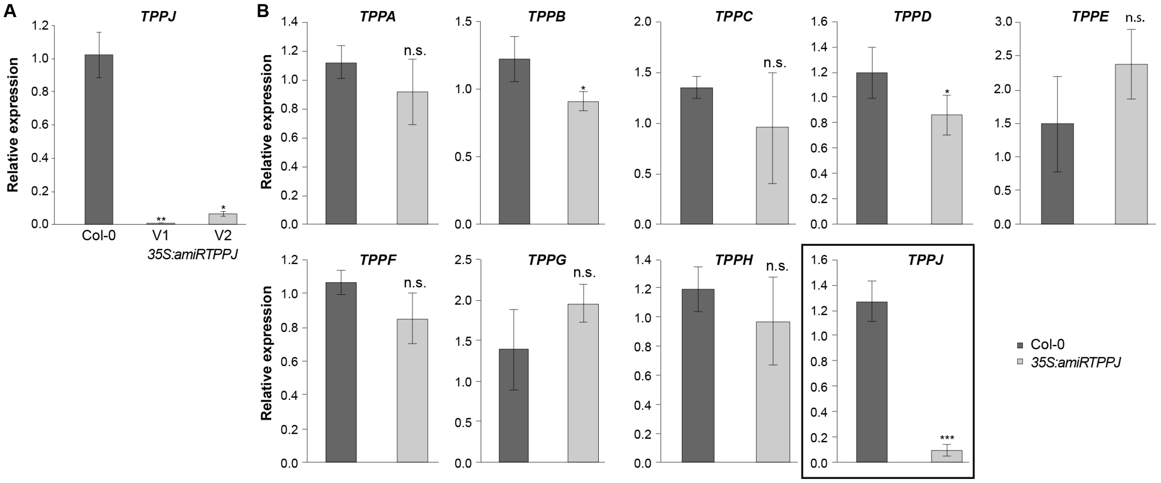

Supplementary Figure S15. Expression of *TPP* genes in *35S:amiRTPPJ* plants. (**A**) Downregulation of *TPPJ* expression in *35S:amiRTPPJ* V1 and V2 rosettes shown by RT-qPCR, relative to Col-0. (**B**) Expression of all *TPPs* in long day-grown Col-0 and *35S:amiRTPPJ* V1 rosettes, harvested without roots at 10 days after germination by RT-qPCR. In this experiment *TPPI* was not detectable. Error bars denote s.d.; ***P*<0.01 (one-way ANOVA).

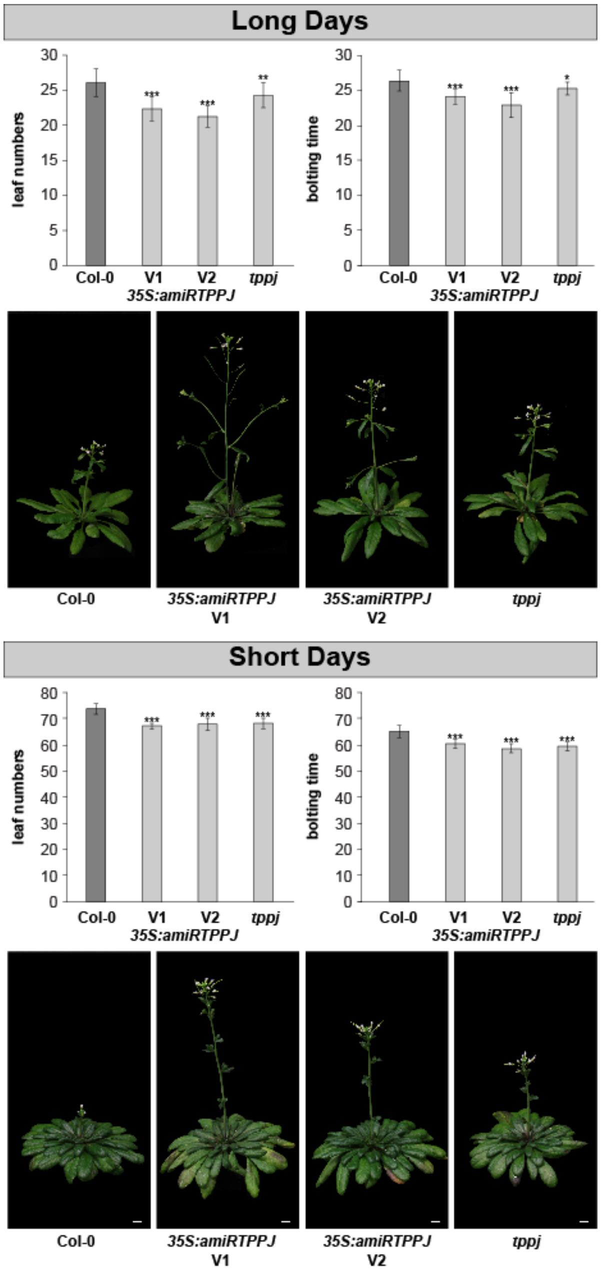

Supplementary Figure S16. Earlier flowering of *35S:amiRTPPJ* and *tppj* lines. Flowering time of *35S:amiRTPPJ* V1 and V2 and *tppj* T-DNA insertion line as shown by the total leaf numbers and days to bolting, relative to the wild type in long days and in short days. V1 and V2 indicate two independent versions of artificial microRNAs designed to target *TPPJ* transcript. Representative pictures of *35S:amiRTPPJ* V1 line with stronger reduction in *TPPJ* expression, *35S:amiRTPPJ V2* and *tppj* in comparison to Col-0 are depicted. Images were digitally extracted for comparison. Scale bars are 1 cm. Error bars denote s.d.; significance calculated based on one way Student's *t*-test; *P<0.05, **P<0.01, ***P<0.001.

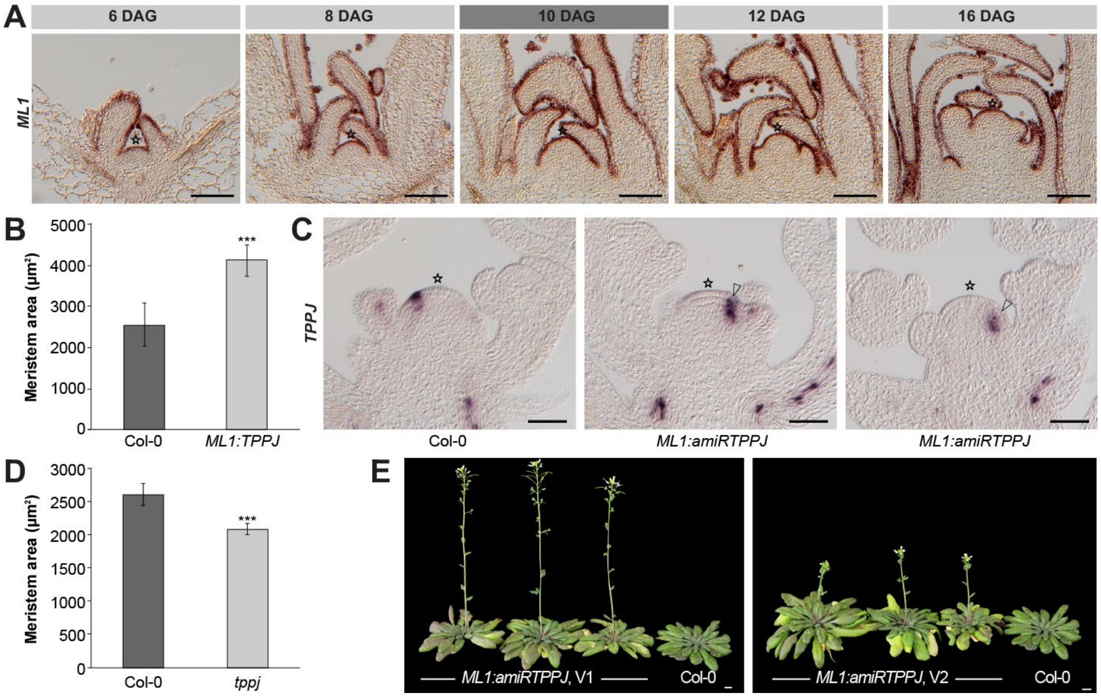

Supplementary Figure S17. Importance of TPPJ in the outer meristem layer. (**A**) RNA *in situ* hybridization using a specific probe against *MERISTEM LAYER1* (*ML1*) on longitudinal sections through Col-0 apices from plants grown in long day. 6, 8 days after germination (DAG) depict vegetative shoot apical meristems (SAMs), 10 DAG the transition to flowering, and 12, 16 DAG inflorescence SAMs. Star indicate SAM summit. (**B**) Meristem area measured from representative single middle sections through apices of *ML1:TPPJ* and Col-0 plants grown in long days, n=9 each. (**C**) RNA *in situ* hybridization with a specific probe against *TPPJ* did not detect *TPPJ* transcript in L1 (arrow head) on longitudinal sections through SAMs of *ML1:amiRTPPJ* plants (V1). Pictures depict two biological replicates of representative inflorescence SAMs of *ML1:amiRTPPJ* in comparison to Col-0. Star indicate SAM summit. (**D**) Meristem area measured from representative single middle sections through apices of *tppj* and Col-0 plants grown in long days, n=10 each. (**E**) Representative pictures of the stronger *ML1:amiRTPPJ V1* line and *ML1:amiRTPPJ V2* in comparison to Col-0 grown in short day conditions indicating the earlier flowering phenotype of the knockdown lines. Please note that the same Col-0 plant was used for comparison in both images. Images were digitally extracted for comparison. Scale bars: 100 µm (A), 50 µm (C), 1 cm (E).

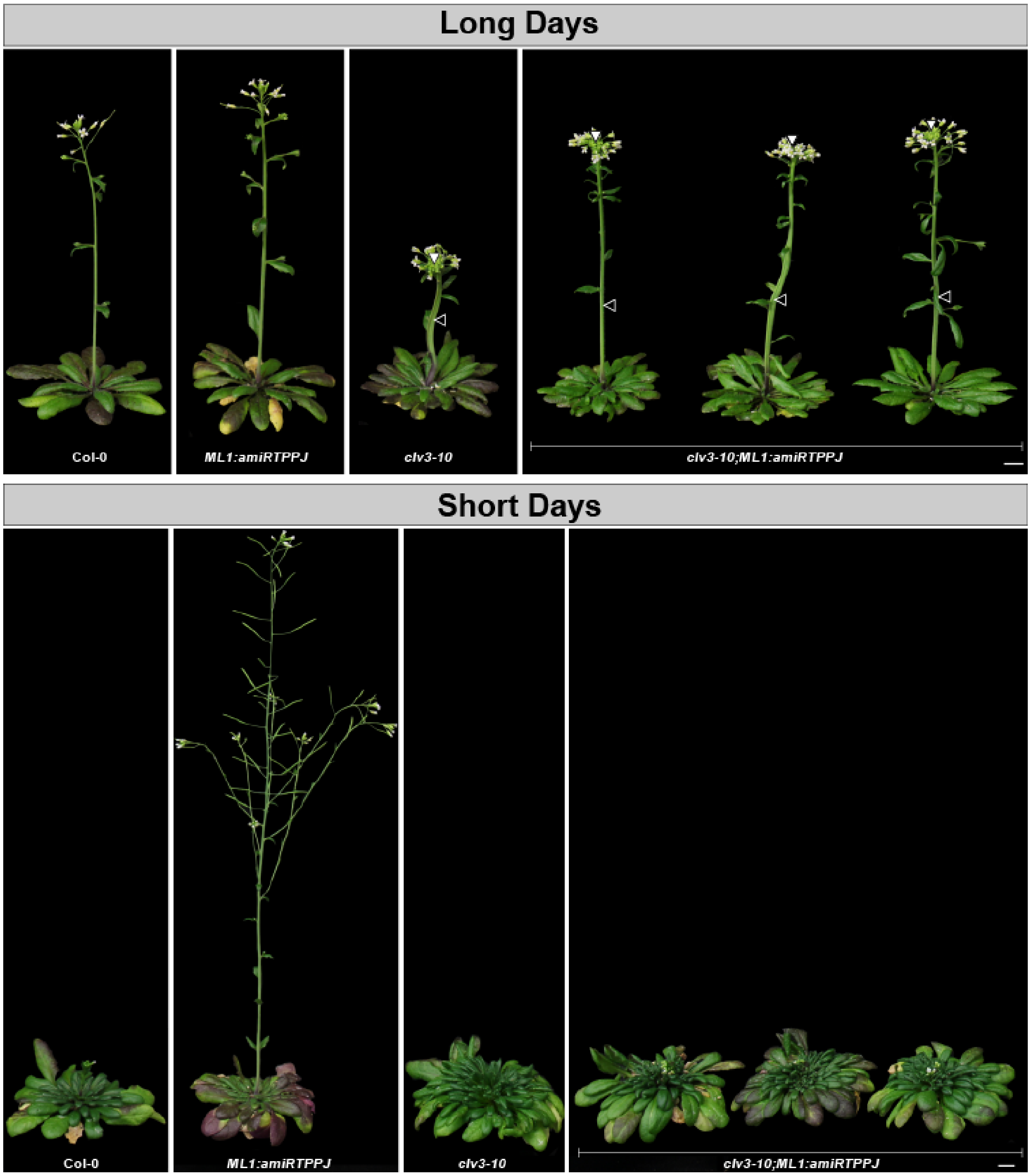

Supplementary Figure S18. Wild-type like flowering phenotype when *ML1:amiRTPPJ* is introgressed into *clv3-10*. Representative pictures of *ML1:amiRTPPJ V1;clv3-10* next to the parental lines and wild type (Col-0) in long days (upper panel) and short (lower panel) indicating a wild-type like flowering phenotype. Open and closed arrowheads indicate fasciated stem and enormous shoot apical meristem, respectively. Note the reduced phenotype of the crosses as compared to the *clv3-10* mutant. Images were digitally extracted for comparison. Scale bars are 1 cm.

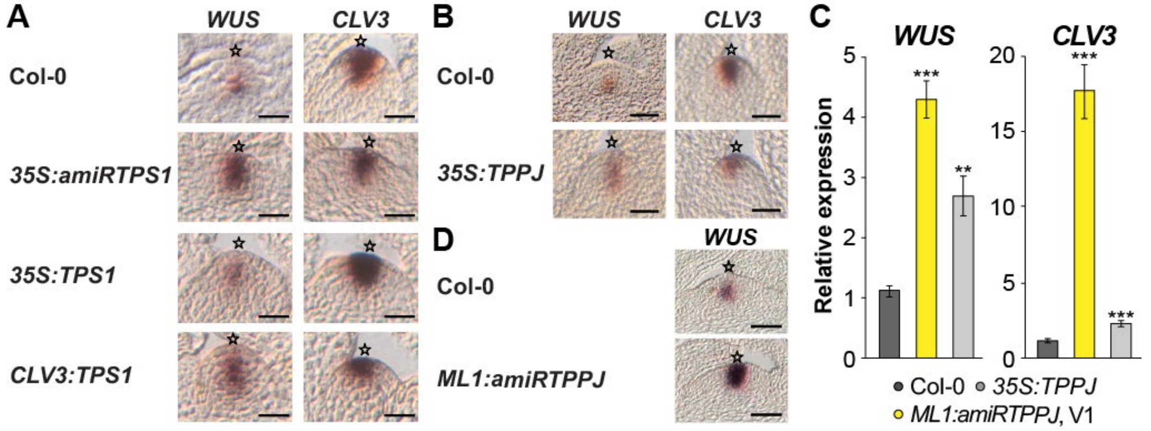

Supplementary Figure S19. *WUS* and *CLV3* expression in apices of trehalose lines. (**A,B**) *WUS* and *CLV3* expression by RNA *in situ* hybridization on (**A**) longitudinal sections through Col-0, *35S:amiRTPS1, 35S:TPS1* and *CLV3:TPS1* and (**B**) Col-0, and *35S:TPPJ* apices of long day-grown plants. (**C**) Relative expression of *WUS* and *CLV3* in short day-grown Col-0, *35S:TPPJ* and *ML1:amiRTPPJ* V1 apices. n=4 (**D**) RNA *in situ* hybridization on longitudinal sections through Col-0, and *ML1:amiRTPPJ* apices. n=3.

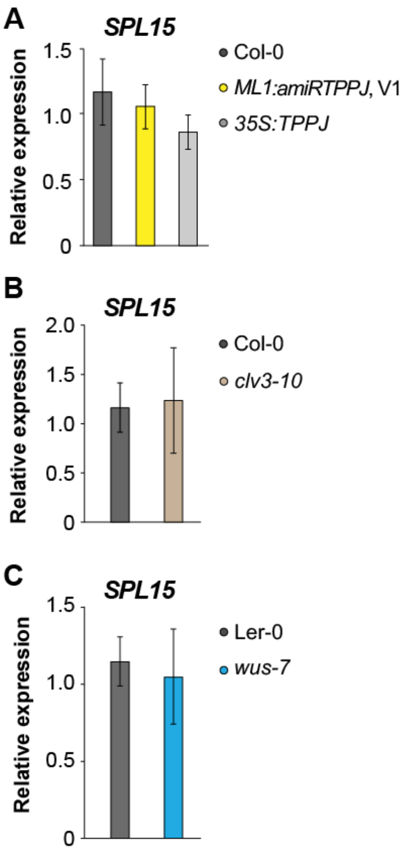

Supplementary Figure S20. Expression of *SPL15* at the shoot apical meristem (SAM). *SPL15* does not significantly change at the SAM of (**A**) *ML1:amiRTPPJ* and *35S:TPPJ,* (**B**) *clv3-10*, and (**C**) *wus-7* compared to its expression in the corresponding wild type. Statistical significance was calculated using a one-way ANOVA. Error bars denote s.d.

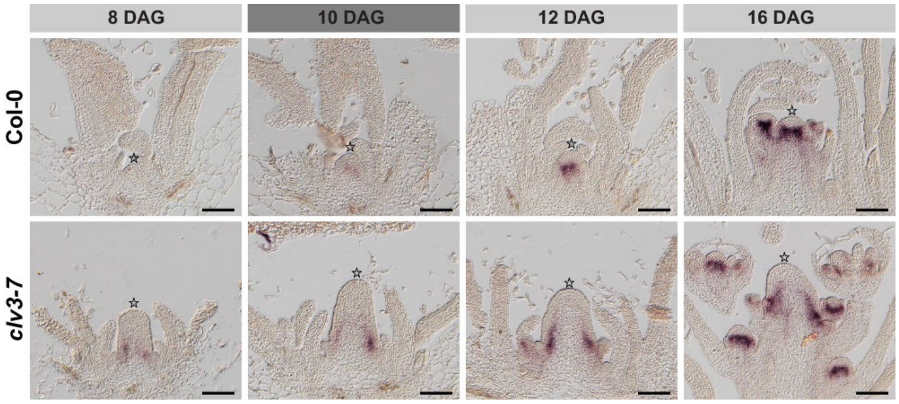

Supplementary Figure S21. *SPL4* expression in *clv3* apices. RNA *in situ* hybridization using an *SPL4* probe on apices of long days grown Col-0 and *clv3-7* mutant plants. Note the earlier expression of *SPL4* in *clv3* apices at 8 days after germination (DAG). Stars indicate SAM summit. Scale bars: 100μm.

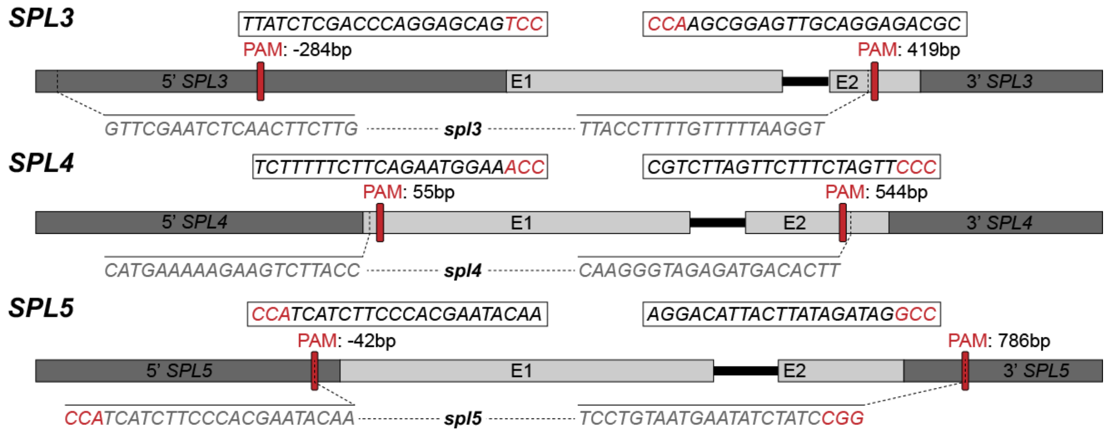

Supplementary Figure S22. *SPL3, SPL4,* and *SPL5* mutant lines. Schematic illustration of the deletions in the *SPL3* (At2g33810), *SPL4* (At1g53160), and *SPL5* (At3g15270) locus generated by CRISPR/*Cas9*. Positions of the PAM sequence are given relative to the transcription start site (ATG). Sequences of guided RNAs with PAM sites (in red) are given in white boxes. In grey the sequences flanking the deletion are presented. E – exon.

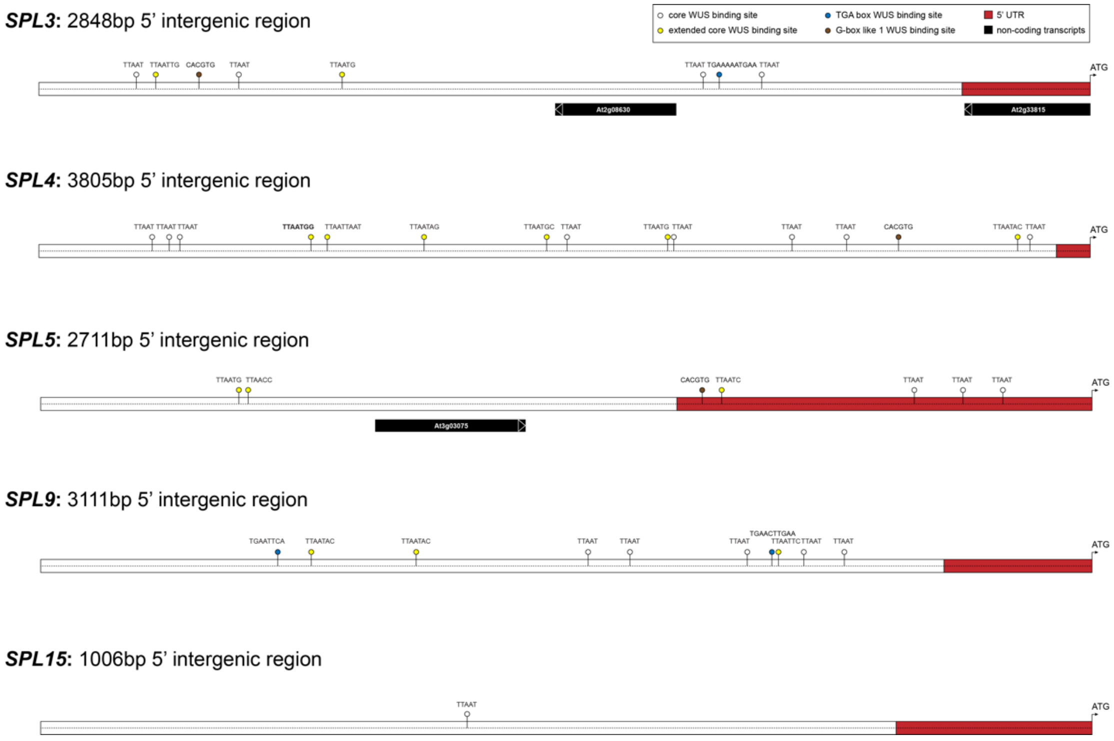

Supplementary Figure S23. Potential *SPL*^{WUS} sites in the 5' intergenic regions of *SPL3*, *SPL4*, *SPL5*, *SPL9*, and *SPL15*. Overview of analyses of *SPL3*, *SPL4, SPL5, SPL9* and *SPL15* 5' intergenic sequences for the presence of putative core binding sites (white circles), extended binding sites (yellow circles), TGA boxes (green circles) and G-box-like sites (brown circles). Red boxes indicate *5'UTR*s. Black boxes represent putative transcripts annotated in the intergenic regions upstream the ATG of *SPL3* and *SPL5.* The total sequence lengths are given in base pairs (bp).

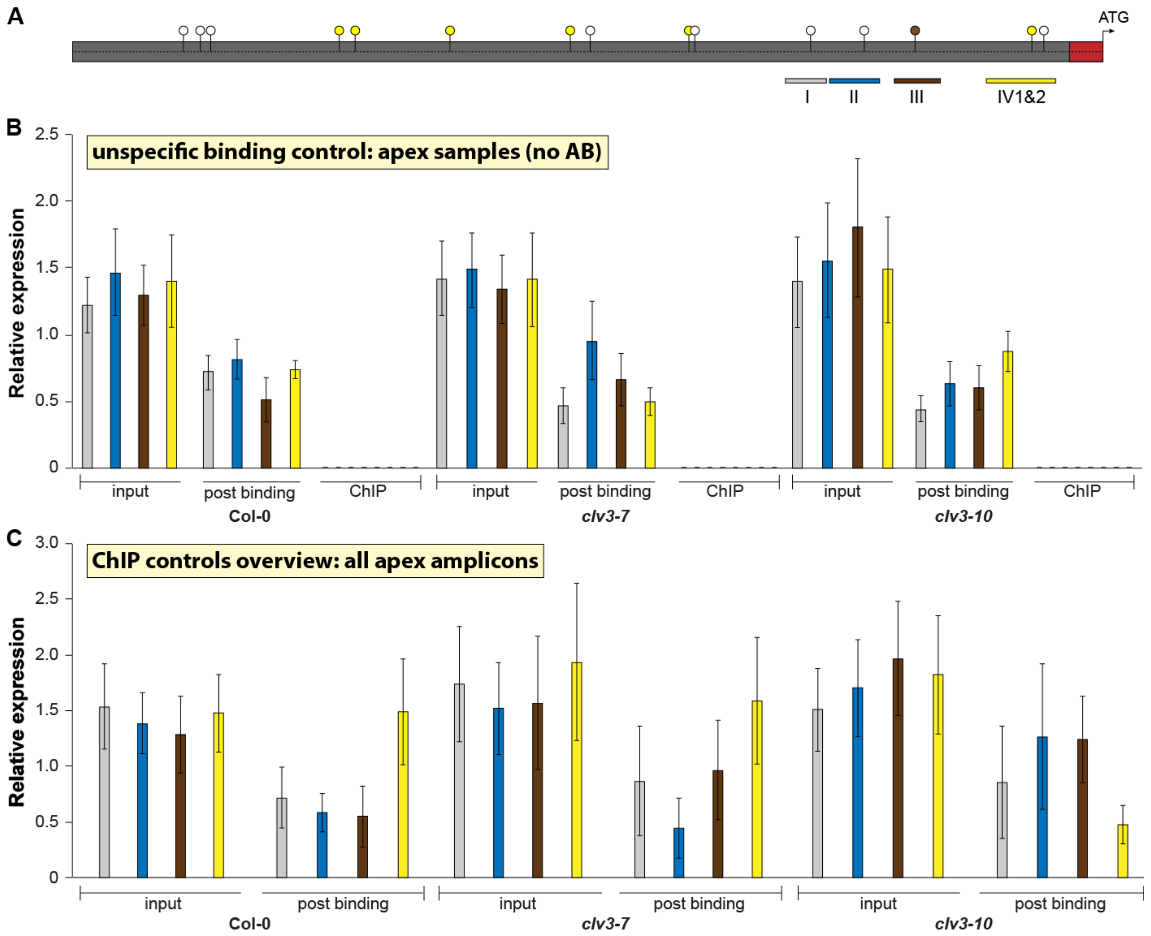

Supplementary Figure S24. ChIP-PCR control experiments. (**A**) Overview of 5' *SPL4* intergenic sequence with all investigated putative *SPL4*^{WUS} sites (white, yellow and brown circles), position of ChIP-PCR amplicons corresponding to the results shown in (B and C). Black framed boxes marked with **I**, **II**, **III**, and **VI** (1&2) indicate *5'SPL4* regions directly bound by WUS – **I**: -1073 – -1068 bp, **II**: -880 –-875 bp, **III**: -697 – -691 bp, and IV: -259 – -209, as presented in Figure 5H. (**B,C**) Relative expression of investigated regions (I-IV) containing *SPL4*^{WUS} elements in (**B**) Col-0, *clv3-7* and *clv3-10* shoot apex samples without WUS antibody. (**C**) Control overview of all investigated putative *SPL4*^{WUS} sites in input and post binding fraction samples. Error bars denote s.d.

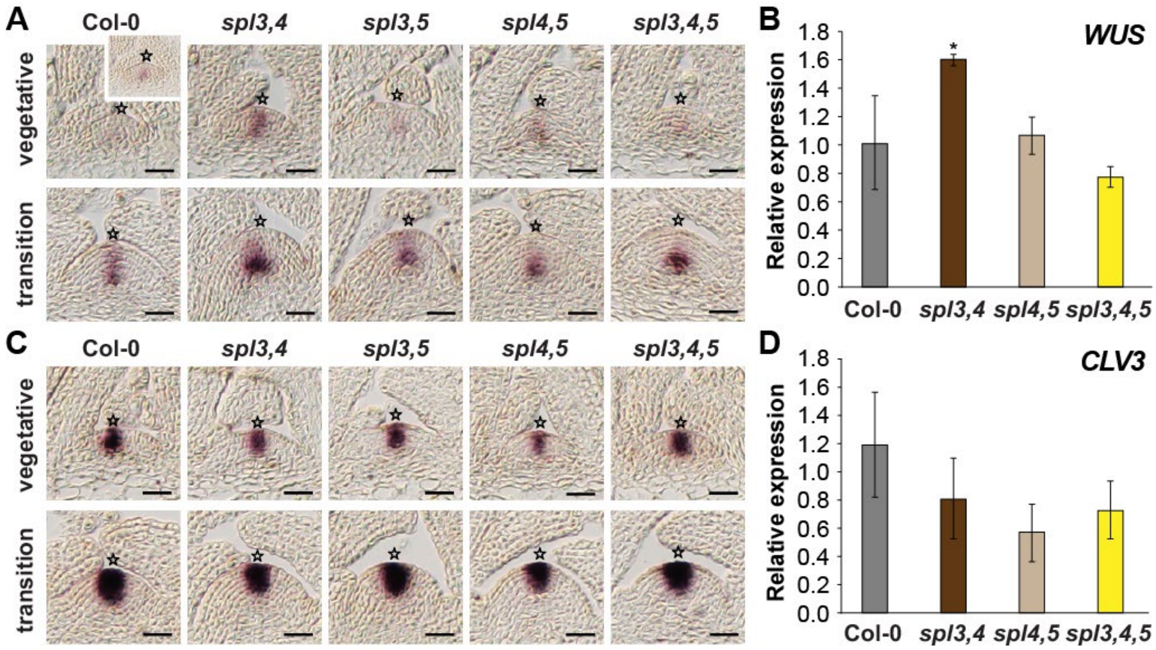

Supplementary Figure S25. *WUS* and *CLV3* expression in apices of short day-grown SPL double and triple CRISPR/*Cas9* deletion mutants. Expression provided as (**A,C**) RNA *in situ* hybridization and (**B,D**) RT-qPCR. *WUS* (A,B) and *CLV3* (C,D). n=3. Inset in A illustrates staining on Col-0 sections after longer incubation time. Error bars denote s.d.; significance calculated based on a one-way ANOVA; $*P<0.05$. Scale bars: 25µm. Star indicates shoot apical meristem summit.

Supplementary Table S1. Flowering times of Col-0, mutants and transgenic plants. DTF, days to flowering (equals bolting time, see before); RLN, rosette leaf number; CLN, cauline leaf number; TLN, total leaf number; n, number of individual plants; p-value of student's *t*-test for DTF with Co-0; LD, long days; SD, short days; NA, not analyzed (leaf number above 200). Please note that data marked with an asterisk (*) were obtained from the same experiment.

	DTF	DTF range	RLN	CLN	TLN	TLN range	n	p-value
**Presented in Figures 4 and 5 of the main text:**								
**Experiment 1 (SD)**								
Col-0	71.6	69-75	66.4	11.5	77.9	76-82	20	-
ML1:amiRTPPJ V1 #1	55.0	50-58	49.4	12.0	61.3	58-63	20	3.29E-22
ML1:amiRTPPJ V2 #5	64.4	61-66	52.8	9.2	62.0	60-64	20	3.07E-14
**Experiment 3 (SD)***								
Col-0	82.2	79-86	68.6	11.9	80.5	76-86	22	-
ML1:amiRTPPJ V1 #1	65.3	61-69	56.7	9.9	66.5	63-72	22	1.08E-25
clv3-10	95.8	95-101	NA	NA	NA	NA	26	2.17E-25
clv3-10;ML1:amiRTPPJ V1 #1_1	87.3	84-92	NA	NA	NA	NA	24	4.39E-09
clv3-10;ML1:amiRTPPJ V1 #1_3	85.8	82-91	NA	NA	NA	NA	23	4.05E-05
**Experiment 6 (SD)** *								
Col-0	72.4	69-75	67.9	9	77.2	74-81	26	-
spl3	73.4	71-75	68.3	10	78.0	75-81	23	0.15
spl4	74.0	71-77	69.2	14	83.0	80-86	26	5.00E-05
spl5	74.0	71-77	68.0	10	77.8	74-82	24	0.45
spl34	77.9	76-80	74.1	17	90.9	87-94	24	5.04E-09
spl35	73.2	71-75	68.4	10	77.9	75-81	24	0.02
spl45	76.8	74-78	73.0	16	76.8	86-93	24	1.25E-11
spl345	84.1	82-87	79.6	19	99.0	94-112	27	2.79E-26
**Only presented as supplemental material:**								
**Experiment 2 (LD)**								
Col-0	22.8	22-25	12.1	3.2	15.3	14-16	20	-
ML1:amiRTPPJ V1 #1	20.0	17-21	9.0	3.1	12.1	10-13	20	4.33E-10
ML1:amiRTPPJ V2 #5	21.0	20-22	10.3	2.9	13.2	10-14	20	9.95E-06
**Experiment 3 (SD)***								
clv3-7	87.5	85-91	NA	NA	NA	NA	25	1.38E-09
clv3-7;ML1:amiRTPPJ V1 #1_2	74.6	70-78	NA	NA	NA	NA	24	5.50E-22
clv3-7;ML1:amiRTPPJ V1 #1_12	76.9	73-80	NA	NA	NA	NA	24	1.47E-19
**Experiment 4 (LD)**								
Col-0	26.5	25-29	20.6	5,1	25,7	24-29	31	-
35S:amiRTPPJ V1 #1	24.3	22-26	17.8	4.6	22.3	21-25	32	1.16E-08
35S:amiRTPPJ V1 #2	23.0	21-26	16.8	4.3	21.2	19-23	32	2.24E-11
tppj	25.8	24-28	19.8	4.8	24.6	22-27	35	0.04
**Experiment 5 (SD)**								
Col-0	65.1	61-68	63.7	10.1	73.7	70-76	30	-
35S:amiRTPPJ V1 #1	60.4	57-62	58.4	9.7	68,1	63-71	30	6.63E-12
35S:amiRTPPJ V1 #2	58.3	56-60	57.8	9.5	67.2	66-69	30	1.47E-17
tppj	59.3	57-62	58.4	9.4	67.8	65-72	30	6.15E-15
**Experiment 6 (SD)***								
spl345 (Xie *et al*, 2020)	83.2	80-86	78.6	18.3	96.6	92-101	25	2.46E-20

Supplementary Table S2. Oligonucleotides for constructs used for the generation of transgenic lines and for transactivation assays.

Gene (AGI)	Oligo	Construct	Sequence (5'→ 3')
TPPJ At5g65140	P-0188	pVW501 *amiRTPPJ V2*	gaTATAATATATCCCCAAGTCGAtctctcttttgtattcc
	P-0189		gaTCGACTTGGGGATATATTATAtcaaagagaatcaatga
	P-0190		gaTCAACTTGGGGATTTATTATTtcacaggtcgtgatatg
	P-0191		gaAATAATAAATCCCCAAGTTGAtctacatatatattcct
TPPJ At5g65140	P-0192	pVW502 *amiRTPPJ V2*	gaTCTAAATGTACTTCACTGCCCtctctcttttgtattcc
	P-0193		gaGGGCAGTGAAGTACATTTAGAtcaaagagaatcaatga
	P-0194		gaGGACAGTGAAGTAGATTTAGTtcacaggtcgtgatatg
	P-0195		gaACTAAATCTACTTCACTGTCCtctacatatatattcct
TPPJ At5g65140	G-8641	pVW279 *35S:TPPJ*	ATGGGTTATGATAATGTATGTG
	G-8642		TTAAACGTTTGGCTTCTTGACAC
5' TPPJ At5g65140	P-1620	pMML049 *5' TPPJ (#1)*	aagggtaccaGAAATACCCTCAAAGCAAATTTGA
	P-1659		CCGTGGCGCGCCCGGTCTTggcgtct
	P-1621	pMML050 *5' TPPJ (#2)*	attggtaccacaGAGTCCACAAGTTTAACAAACAACAGA
	P-1659		CCGTGGCGCGCCCGGTCTTggcgtct
	P-1654	pMML051 *5' TPPJ (#3)*	cttggtaccTTGTCATCATTAAGATGGGATGACA
	P-1659		CCGTGGCGCGCCCGGTCTTggcgtct
	P-1655	pMML052 *5' TPPJ (#4)*	tatggtaccAACGTGGGTGATCATCTCTCG
	P-1659		CCGTGGCGCGCCCGGTCTTggcgtct
	P-1657	pMML054 *5' TPPJ (#5)*	ctaggtaccGTGATTATTGTCAAGGCACCTTAT
	P-1659		CCGTGGCGCGCCCGGTCTTggcgtct
	P-1658	pMML055 *5' TPPJ (#6)*	ttaggtaccCCAAACGAAACAAAAACG
	P-1659		CCGTGGCGCGCCCGGTCTTggcgtct
5' SPL4 At1g53160	P-2040	pMML070 *5'SPL4 (#1)*	ATATGGTACCGCATAAATCCAGATGTTCCTCC
	P-0243		ATATACTAGTCCTTGTCCTTGTGATCTCTTACC
	P-2041	pMML071 *5'SPL4 (#2)*	ATATGGTACCGCTACGGACAAGGTCCTACG
	P-2043		ATATACTAGTCCTTGTCCTTGTGATCTCTTACC
	P-2042	pMML072 *5'SPL4 (#3)*	ATATGGTACCGGCAGCTTTTGGCTTTGG
	P-2043		ATATACTAGTCCTTGTCCTTGTGATCTCTTACC
5' WUS At2g17950	P-2040	pMML067 *5'WUS*	ATATGGTACCGCATAAATCCAGATGTTCCTCC
	P-0243		ATATACTAGTCCTTGTCCTTGTGATCTCTTACC
	P-2041	pMML088 *5'WUS* mutated*	CATGTTCATAA**TTTT**ACCTGTCTTCACAC
	P-2043		GTGTGAAGACAGGT**AAAA**TTATGAACATG
SPL3 At2g33810	P-1334	pMML040 *CRISPR Cas9 SPL3*	accaggtctcaattgAATAGAGCTGGGTCCTCGTCgttttagagctagaaatagcaag
	P-1336		tggtggtctctaaacAGCGGAGTTGCAGGAGACGCcaatctcttagtcgactctacc
SPL4 At1g53160	P-1340	pMMl043 *CRISPR Cas9 SPL4*	accaggtctcaattgGAAAAAGAAGTCTTACCTTGgttttagagctagaaatagcaag
	P-1341		tggtggtctctaaacTTGATCTTTCTTGATTCTGCcaatctcttagtcgactctacc
SPL5 At3g15270	P-1343	pMML027 *CRISPR Cas9 SPL4*	accaggtctcaattgTTGTATTCGTGGGAAGATGAgttttagagctagaaatagcaag
	P-1345		tggtggtctctaaacGATAGATATTCATTACAGGAcaatctcttagtcgactctacc

* Mutagenesis PCR was performed on the pMML067 plasmid. The resulting mutated promoter was sequenced after.

Supplementary Table S3. Oligonucleotides used for genotyping.

Transgenic line	Oligo	Sequence (5'→ 3')	Position
VW507	P-0110	AGTAGTATGAATTTTCTCTCAC	*ML1 promoter*
ML1:amiRTPPJ (V1)	P-0189	*gaTCGACTTGGGGATATATTATAtcaaagagaatcaatga*	*miRNA stemloop*
VW508	P-0110	AGTAGTATGAATTTTCTCTCAC	*ML1 promoter*
ML1:amiRTPPJ (V2)	P-0193	*gaGGGCAGTGAAGTACATTTAGAtcaaagagaatcaatga*	*miRNA stemloop*
VW504	P-0107	AACCTCCTCGGATTCCATTGCC	*CaMV 35S promoter*
35S:amiRTPPJ (V1)	P-0189	*gaTCGACTTGGGGATATATTATAtcaaagagaatcaatga*	*miRNA stemloop*
VW505	P-0107	AACCTCCTCGGATTCCATTGCC	*CaMV 35S promoter*
35S:amiRTPPJ (V2)	P-0193	*gaGGGCAGTGAAGTACATTTAGAtcaaagagaatcaatga*	*miRNA stemloop*
VW161	P-0107	AACCTCCTCGGATTCCATTGCC	*CaMV 35S promoter*
35S:TPPJ	P-0121	ATTGTGTTCTTCTTCGGAAGAG	*TPPJ* CDS
VW161	P-0107	AACCTCCTCGGATTCCATTGCC	*CaMV 35S promoter*
35S:TPS1	P-0120	ATTATAAGGCCTAACTTCTTGC	*TPS1* CDS
tppj	P-0208	TGACCAGCCGCGCTGAATGTG	*GABI Kat LB*
GABI_215C08	P-1300	GATGCATCGACAAGGTTTATAGCTTTG	*TPPJ forward*
	P-1302	GTTACAGTTTTCAAATCCGAGTGACTCTA	*TPPJ reverse*
*clv3-10**	P-1386	TGATGCTTCTGATCTCACTCAAGC	*CLV3 forward*
	P-1387	CCATTTGCTCCAACCCATTC	*CLV3 reverse*
clv3-7	P-1519	CCTGAGGTTGGGCAGAAACA	*En-1* LB
	P-1518	TCAAGCTCATGCTCACGTTCA	*CLV3 forward*
	P-1387	CCATTTGCTCCAACCCATTC	*CLV3 reverse*
spl3	P-1476	GTGGTGTCATAGCAT	*5'SPL3*
	P-1113	TGGGAATCAAACGTGACTAGG	*3'SPL3*
spl4	P-1498	GCTTAACCTATTAGAGTTTC	*5'SPL4*
	P-1499	TCGTGGCTCTGAAACTTCAG	*3'SPL4*
spl5	P-1497	GGAAGCAAGCACATCACGTA	*5'SPL4*
	P-1475	CGCAAATAAGAATCAAGTCGAGa	*3'SPL4*
Cas9	P-1478	CGACCCTCATCCACCAGTCGATTAC	*Cas9* cassette
	P-1480	CCCAGGCTTTACACTTTATGCTTCC	

*Note that *clv3-10* was genotyped based on sequence analyses.

Supplementary Table S4. Oligonucleotides used for RT-qPCR.

Gene	Oligo	Sequence (5'→3')
TPPJ	P-1300	GATGCATCGACAAGGTTTATAGCTTTG
At5g65140	P-1301	TGAGAAACCTTTAGTTGGGCCTTT
TPPA	P-1493	GATGATCGATAAGGTGCTTTGTTC
At5g51460	P-1494	GCGGTAATGCACAGAGATGC
TPPB	P-1489	CAAGGTAGAAAGGTACTTGAAATCCG
At1g78090	P-1490	ACACCGGCACAACATCATCC
TPPC	P-1487	CAAGGCAGAAAGGTCTTAGAACTCC
At1g22210	P-1488	TCCCTAATGAGTTTAGCAAAAATTCG
TPPD	P-1483	GATGCCGTGATAAGGTTCGTAGAT
At1g35910	P-1484	TGGAAGGTCCTTTGATGTCCA
TPPE	P-1485	CAAGGAAGAAAAGTATTGGAGATTCG
At2g22190	P-1486	ATAGGAAAAACATCGGTACAATTATCA
TPPF	P-1481	ACGCGGGTAGTCATGGAATG
At4g12430	P-1482	CACTTCCTTACCCTGTTGGTCAGT
TPPG	P-1303	GTAAATCCAAATGGATCCCCTGAAG
At4g22590	P-1304	TTACCTCCTCACCCTGTTGGTC
TPPH	P-1495	AGAGTCACTTGGATACGCTAATTGTAC
At4g39770	P-1496	GCCAAGACCTTGTCTTCGTTC
TPPI	P-1482	GCCTTCGCCTCTGTCTCGTA
At5g10100	P-1483	GATGATCGATAAGGTGCTTTGTTC
SPL3	P-1617	GTTTCATGCCAAAGCTCCTC
At2g33810	P-1618	TTTTCCGCCTTCTCTCGTT
SPL4	P-0546	AATGGTCAGGTGGTGATGCAG
At1g53160	P-0547	GCATAGGAAGTGTCATCTCTACCCTT
SPL5	P-0548	CAGCAGGTTTCATGAGCTACCAG
At3g15270	P-0549	CAAAACTGTCACCAGAGATCTTCCTC
SPL9	P-1307	CTTCGCTTTACGAAAATGGTGATG
At2g42200	P-1308	ACTGGCCGCCTCATCACTCT
SPL15	P-1309	CATCTCTTTACGGAAACCCCAATG
At3g57920	P-1310	GCCGCTGCATCACTGATCTT
**mir156**	P-0342	GCGGCGGTGACAGAAGAGAGT
miRNA universal	P-0343	GTGCAGGGTCCGAGGT
CLV3	P-1386	TGATGCTTCTGATCTCACTCAAGC
At2g27250	P-1387	CCATTTGCTCCAACCCATTC
WUS	P-1382	CCCAGCTTCAATAACGGGAAT
At2g17950	P-1383	ACAGTCTTGTTCCATAGATCCATAGAC
UBQ10	P-0348	CACACTCCACTTGGTCTTGCGT
At4g05320	P-0349	TGGTCTTTCCGGTGAGAGTCTTCA
TUB2	P-0344	GAGCCTTACAACGCTACTCTGTCTGTC
At5g62690	P-0345	ACACCAGACATAGTAGCAGAAATCAAG
SAND	P-0346	AACTCTATGCAGCATTTGATCCACT
At2g28390	P-0347	TGATTGCATATCTTTATCGCCATC
PDF2	P-0350	TAACGTGGCCAAAATGATGC
At1g13320	P-0351	GTTCTCCACAACCGCTTGGT
GAPDH* At1g13440	P-1125	TCTCGATCTCAATTTCGCAAAA
	P-1126	CGAAACCGTTGATTCCGATTC
	P-1127	TTGGTGACAACAGGTCAAGCA
	P-1128	AAACTTGTCGCTCAATGCAATC
**miR156** stem-loop **	P-1010	GTCGTATCCAGTGCAGGGTCCGAGGTATTCGCACTGGATACGACG

* Primers used for cDNA quality check according to (Czechowski *et al*, 2004).

** Please note that this oligo was used for cDNA synthesis according to (Varkonyi-Gasic *et al*, 2007).

Supplementary Table S5. Oligonucleotides used for ChIP-PCR.

Gene	Oligo	Region	Sequence (5'→3')
	P-1624<br>P-1625	**I**:<br>-3073 bp -2941 bp	GAAATACCCTCAAAGCAAATTTGA<br>AGCTTCTAAAATTCATCAAAACCAA
	P-1628<br>P-1783	**II (1)**:<br>-2150 bp -1924 bp	GAGTCCACAAGTTTAACAAACAACAGA<br>TCCCATCTTAATGATGACAAACA
	P-1626<br>P-1782	**II (2)**:<br>-1943 bp -1847 bp	TTGTCATCATTAAGATGGGATGACA<br>TACTGATATATGTTTGCAAAGTTT
	P-1784<br>P-1627	**II (3+4)**:<br>-1887 bp -1751 bp	TGAATGGAGATAATATAAAACTTTGC<br>TCGAAGACGCCCCTTGTC
	P-1629<br>P-1630	**II (5)**<br>-1818 bp – 1617 bp	GCCGCTGCTGGTTTTGAG<br>TGTCATTGCATGCATGTGTG
	P-1628<br>P-1782	**II:**<br>-2150 bp -1847 bp	GAGTCCACAAGTTTAACAAACAACAGA<br>TACTGATATATGTTTGCAAAGTTT
	P-1636<br>P-1785	**III (1)**:<br>-697 bp -604 bp	CGTGTTCCTATCCATGTTCCT<br>GTATAATGATAAATTTGTAGTAATACC
	P-1786<br>P-1637	**III (2+3)**:<br>-630 bp -521 bp	GGTATTACTACAAATTTATCATTATAC<br>CCACTTCAACGTTATATCAACCG
5' TPPJ: At5g65140	P-1640<br>P-1641	**III (4)**<br>-826 bp -663 bp	TGTGATTATTGTCAAGGCACCTTAT<br>TTTCATGATTGTATAGGAACATGG
	P-1638<br>P-1639	**III (5)**<br>-561 bp – 381 bp	CCCCAAACGAAACAAAAACG<br>CCCCGAGCCAAAGGAAAA
	P-1636<br>P-1637	**III:**<br>-697 bp -521 bp	CGTGTTCCTATCCATGTTCCT<br>CCACTTCAACGTTATATCAACCG
	P-1631<br>P-1632	**IV (1):**<br>-1353bp -1167 bp	AACGTGGGTGATCATCTCTCG<br>CGTTCAATAGTTGGCACTAAACCA
	P-1633<br>P-1635	**IV (2):**<br>-1184 bp - 891bp	AGTGCCAACTATTGAACGATTG<br>CGATGCATGCAGCCACAC
	P-1633<br>P-1634	**IV (3):**<br>-1185 bp – 968 bp	AGTGCCAACTATTGAACGATTG<br>CCATCACGCTCTCGAACTTTCT
	P-1642<br>P-1643	**V:**<br>-94 bp – 44 bp	TCCTCCTCCTTCCTAGTTTCTCG<br>ATCCCGGTCTTGGCGTCT
	P-2014<br>P-2015	**VI (1):**<br>-1513 bp – 1387 bp	CCAAAAGGGGACGCGAG<br>TCGATTCCGGTTTCATCGTT
	P-2016<br>P-2017	**VI (2):**<br>-1513 bp – 1387bp	CGAATACGACGTAGACGAATAAGACA<br>AGCCATGGGACCGAGGTT
	P-2109<br>P-2110	**I**:<br>-1166 bp -1015 bp	CTTATCTGTTTCCTGCTTTGTTGG<br>CAGTGTGTTGTGTGGAGAAGCA
5' SPL4: At1g53160	P-2111<br>P-2112	**II**:<br>-1005 bp -821 bp	AGGAAGGTAAAAGTGAGAGGCTTTT<br>GGAGGAATTACAGATTGAAAGATGG
	P-2113<br>P-2114	**III**:<br>-765 bp -593 bp	GAAAGAAATCAAAAGGGTTAGATCGT<br>TTTGGTAGACTCTTATTGGAAATGCA
	P-2115<br>P-2116	**IV (1&2):**<br>-425 bp -169 bp	CCTCTTAAAGGCAACCAATGATATC<br>GTGCATGATTACGTTGATGTGC
5' UBQ10: At4g05320	P-1889<br>P-1890	-582 bp – 306 bp	TCGTGTTTATCAACTCAAAGCACAA<br>TGATCACGGTAGAGAGAATTGAGAGA

Abbreviations: I, **II**, **III** (***TPPJ***) and **I, II, III; IV** (***SPL4***) regions positive in ChIP-PCR, other regions – negative

Supplementary Table S6. Oligonucleotides used to prepare constructs for probe synthesis.

Gene	Construct ID	Oligo	Sequence (5'→3')
WUS: At2g17950	pVW310	P-0148<br>P-0149	ATGGAGCCGCCACAGCATCAGCATC<br>CTAGTTCAGACGTAGCTCAAGAGAAG
CLV3: At2g27250	pJL102	P-0150<br>P-0151	TCACTCAGTCACTTTCTCTC<br>GAAAATCATGAGATATAATAG
ML1: At4g21750	pVW510	P-0152<br>P-0153	ATGTATCATCCAAACATGTTCG<br>TTAGGCTCCGTCGCAGGCCAGAG
TPPA: At5g51460	pVW518	P-0214<br>G-13892	ATGGACATGAAATCTGGTCACTCGTC<br>TTAACCCATTGATCTCTTCCATGTC
TPPB: At1g78090	pVW516	P-0211<br>P-0212	ATGACTAACCAGAATGTCATCGTTTC<br>TCACTCTTCTCCCACTGTCTTCCTC
TPPC: At1g22210	pVW515	P-0210<br>G-13888	ATGAAGATTACGGATATTTCCGGAA<br>TTATTCTCCAAGTGTTTGTTTCTTC
TPPD: At1g35910	pVW257	G-8814<br>G-8815	ATGACAAACCATAATGCCTTAATC<br>CTATCTTCCTCTTAGTGACATTTG
TPPE: At2g22190	pVW258	G-8816<br>G-8817	ATGGTTAGATTCATCGAAGAAAATATTAC<br>TCATGCCCCACACCTTGACTGTTTCC
TPPF: At4g12430	pVW227	G-8641<br>G-8642	ATGGATTTAAACTCAAACCACAAATC<br>TCAAAAACCAGTAGAATTCTTCTCC
TPPG: At4g22590	pVW514	G-8639<br>G-8640	ATGGATTTGAATATAAACAAGACG<br>TCAAAAACTTGTTTTTGAACTTTC
TPPH: At4g39770	pVW519	P-0215<br>G-13894	ATGGTTAGATTCATAGAAGAAAACAC<br>TCATGCTCCAGATCTCAATTGTTTCC
TPPI: At5g10100	pVW517	P-0216<br>P-0213	ATGTCAGCTAGTCAAAACATTGTCG<br>TCACATTCTTGGCTGCATTTGTTTCC
TPPJ: At5g65140	pVW228	G-8641<br>G-8642	ATGGGTTATGATAATGTATGTG<br>TTAAACGTTTGGCTTCTTGACAC
H4: At2g28740	pAK029	P-0285<br>P-0286	ATGCTGTTACTTACACTGAGCATG<br>CCAGAAAACCCAGATCTCAAATTG
CYCD3;1: AT4G34160	pVW531	P-0250<br>P-0251	ATGGCGATTCGGAAGGAGGAAG<br>TTATGGAGTGGCTACGATTGCCC
CDKB2;1 AT1G76540	pVW533	P-0258<br>P-0259	TGGACGAGGGAGTTATAGCAGTTTC<br>TTAGAGAGAGGACTTTTCTGGCAG

Supplementary Table S7. EMSA oligonucleotides (probes) for *5'TPPJ* At5g65140

Oligo	Modification	Region	Sequence (5'→3')
P-1777<br>P-1778	5' IR DEY6832	**I**:-2815 bp -2766 bp	ctaaaaattcgtttttttcttaatccggaagataaaaaggttataaaga<br>tctttataaccttttatcttccggattaagaaaaaaaacgaatttttag
P-1779<br>P-1780	none – competitor	**I**:-2815 bp -2766 bp	ctaaaaattcgtttttttcttaatccggaagataaaaaggttataaaga<br>tctttataaccttttatcttccggattaagaaaaaaaacgaatttttag
P-2186<br>P-2187	none –<br>mutated competitor	**I**:-2815 bp -2766 bp	ctaaaaattcgtttttttcttcctccggaagataaaaaggttataaaga<br>tctttataaccttttatcttccggaggaagaaaaaaaacgaatttttag
P-1829<br>P-1730	5' IR DEY682	**II (1)**:-2095 bp -2040 bp	ctattatttttcgctttcaaattaatacacatacaaattaacaaaagta<br>tacttttgttaatttgtatgtgtattaatttgaaagcgaaaaaataatag
P-1841<br>P-1842	none – competitor	**II (1)**:-2095 bp -2040 bp	ctattatttttcgctttcaaattaatacacatacaaattaacaaaagta<br>tacttttgttaatttgtatgtgtattaatttgaaagcgaaaaaataatag
P-2188<br>P-2189	none –<br>mutated competitor	**II (1)**:-2095 bp -2040 bp	ctattatttttcgctttcaaattggtacacatacaaattaacaaaagta<br>tacttttgttaatttgtatgtgtaccaatttgaaagcgaaaaaataatag
P-1831<br>P-1832	5' IR DEY682	**II (2)**:-1922 bp -1878 bp	gacataaaagtcacactgtcaaattaatttgaatatgaatggaga<br>tctccattcatattcaaattaatttgacagtgtgacttttatgtc
P-1843<br>P-1844	none – competitor	**II (2)**:-1922 bp -1878 bp	gacataaaagtcacactgtcaaattaatttgaatatgaatggaga<br>tctccattcatattcaaattaatttgacagtgtgacttttatgtc
P-2190<br>P-2191	none –<br>mutated competitor	**II (2)**:-1922 bp -1878 bp	gacataaaagtcacactgtcaaattggtttggtatggatggaga<br>tctccatccataccaaaccaatttgacagtgtgacttttatgtc
P-1833<br>P-1834	5' IR DEY682	**II (3+4)**:-1863 bp -1814 bp	gcaaacatatatcagtattaattaaatgattaatatgcatttgcagccgc<br>gcggctgcaaatgcatattaatcatttaattaatactgatatatgtttgc
P-1845<br>P-1846	none – competitor	**II (3+4)**:-1863 bp -1814 bp	gcaaacatatatcagtattaattaaatgattaatatgcatttgcagccgc<br>gcggctgcaaatgcatattaatcatttaattaatactgatatatgtttgc
P-2192<br>P-2193	none –<br>mutated competitor	**II (3+4)**:-1863 bp -1814 bp	gcaaacatatatcagtattggttaaatgattggtatgcatttgcagccgc<br>gcggctgcaaatgcataccaatcatttaaccaatactgatatatgtttgc
P-1835<br>P-1836	5' IR DEY682	**III (1)**:-674 bp -625 bp	acaatcatgaaagcttttggttttaatctttttattttatttttggtatt<br>aataccaaaaataaaataaaaagattaaaaccaaaagctttcatgattgt
P-1847<br>p-1848	none – competitor	**III (1)**:-674 bp -625 bp	acaatcatgaaagcttttggttttaatctttttattttatttttggtatt<br>aataccaaaaataaaataaaaagattaaaaccaaaagctttcatgattgt
P-2194<br>P-2195	none –<br>mutated competitor	**III (1)**:-674 bp -625 bp	acaatcatgaaagcttttggttttggtctttttattttatttttggtatt<br>aataccaaaaataaaataaaaagaccaaaaccaaaagctttcatgattgt
P-1837<br>P-1838	5' IR DEY682	**III (2)**:-618 bp -569 bp	aatttatcattatacaactgtacataataatagtagtcatcactatacaa<br>ttgtatagtgatgactactattattatgtacagttgtataatgataaatt
P-1849<br>p-1850	none – competitor	**III (2)**:-618 bp -569 bp	aatttatcattatacaactgtacataataatagtagtcatcactatacaa<br>ttgtatagtgatgactactattattatgtacagttgtataatgataaatt
P-2196<br>P-2197	none –<br>mutated competitor	**III (2)**:-618 bp -569 bp	aatttatcattatacaactgtacatggtggtagtagtcatcactatacaa<br>ttgtatagtgatgactactaccaccatgtacagttgtataatgataaatt
P-1839<br>P-1840	5' IR DEY682	**III (3)**:-585 bp -536 bp	tagtcatcactatacaattaatttccccaaacgaaacaaaaacggttgat<br>atcaaccgtttttgtttcgtttggggaaattaattgtatagtgatgacta
P-1851<br>P-1852	none – competitor	**III (3)**:-585 bp -536 bp	tagtcatcactatacaattaatttccccaaacgaaacaaaaacggttgat<br>atcaaccgtttttgtttcgtttggggaaattaattgtatagtgatgacta
P-2198<br>P-2199	none –<br>mutated competitor	**III (3)**:-585 bp -536 bp	tagtcatcactatacaattggtttccccaaacgaaacaaaaacggttgat<br>atcaaccgtttttgtttcgtttggggaaaccaattgtatagtgatgacta

Abbreviations: I, **II**, **III** regions positive in ChIP-PCR.

Supplementary Table S8. EMSA oligonucleotides (probes) for 5'*WUS* At2g17950.

Oligo	Modification	Region	Sequence (5'→3')
P-2172<br>P-2173	5' IR DEY6832	-1088 bp -1038 bp	gaatactcaacatgttcataagtacacctgtcttcacactcgtttcacac<br>gtgtgaaacgagtgtgaagacaggtgtacttatgaacatgttgagtattc
P-2174<br>P-2175	none – competitor	-1088 bp -1038 bp	gaatactcaacatgttcataagtacacctgtcttcacactcgtttcacac<br>gtgtgaaacgagtgtgaagacaggtgtacttatgaacatgttgagtattc
P-2180<br>P-2181	none –<br>mutated competitor	-1088 bp -1038 bp	gaatactcaacatgttcataattttacctgtcttcacactcgtttcacac<br>gtgtgaaacgagtgtgaagacaggtaaaattatgaacatgttgagtattc

SI References

Busch W, Miotk A, Ariel FD, Zhao Z, Forner J, Daum G, Suzaki T, Schuster C, Schultheiss SJ, Leibfried A *et al* (2010) Transcriptional control of a plant stem cell niche. *Dev Cell* 18: 849-861
Czechowski T, Bari RP, Stitt M, Scheible WR, Udvardi MK (2004) Real-time RT-PCR profiling of over 1400 Arabidopsis transcription factors: unprecedented sensitivity reveals novel root- and shoot-specific genes. *Plant J* 38: 366-379

Forner J, Pfeiffer A, Langenecker T, Manavella PA, Lohmann JU (2015) Germline-transmitted genome editing in Arabidopsis thaliana Using TAL-effector-nucleases. *PLoS One* 10: e0121056
Leibfried A, To JP, Busch W, Stehling S, Kehle A, Demar M, Kieber JJ, Lohmann JU (2005) WUSCHEL controls meristem function by direct regulation of cytokinin-inducible response regulators. *Nature* 438: 1172-1175

Schmid M, Davison TS, Henz SR, Pape UJ, Demar M, Vingron M, Scholkopf B, Weigel D, Lohmann JU (2005) A gene expression map of Arabidopsis thaliana development. *Nat Genet* 37: 501-506

Varkonyi-Gasic E, Wu R, Wood M, Walton EF, Hellens RP (2007) Protocol: a highly sensitive RT-PCR method for detection and quantification of microRNAs. *Plant Methods* 3: 12

Wahl V, Ponnu J, Schlereth A, Arrivault S, Langenecker T, Franke A, Feil R, Lunn JE, Stitt M, Schmid M (2013) Regulation of flowering by trehalose-6-phosphate signaling in Arabidopsis thaliana. *Science (New York, NY)* 339: 704-707

Xie Y, Zhou Q, Zhao Y, Li Q, Liu Y, Ma M, Wang B, Shen R, Zheng Z, Wang H (2020) FHY3 and FAR1 Integrate Light Signals with the miR156-SPL Module-Mediated Aging Pathway to Regulate Arabidopsis Flowering. *Mol Plant* 13: 483-498

Yadav RK, Perales M, Gruel J, Girke T, Jonsson H, Reddy GV (2011) WUSCHEL protein movement mediates stem cell homeostasis in the Arabidopsis shoot apex. *Genes Dev* 25: 2025-2030

Zurcher E, Tavor-Deslex D, Lituiev D, Enkerli K, Tarr PT, Muller B (2013) A robust and sensitive synthetic sensor to monitor the transcriptional output of the cytokinin signaling network in planta. *Plant Physiol* 161: 1066-1075
