# Supplementary figures and images for: The trehalose 6-phosphate pathway coordinates dynamic changes at the shoot apical meristem in Arabidopsis thaliana
Source: Plant Physiol. 2025 Jul 10;199(1):kiaf300. doi: 10.1093/plphys/kiaf300 (PMC12415856; doi:10.1093/plphys/kiaf300)

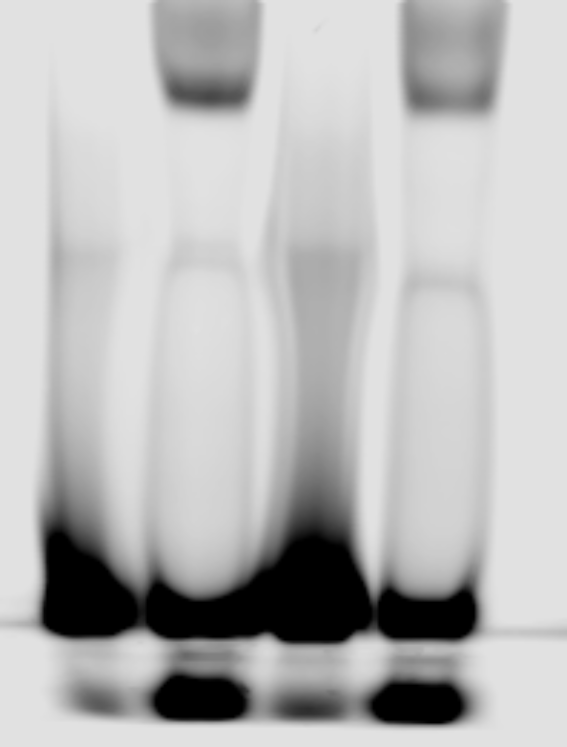

Supplement: kiaf300_Supplementary_Data [file kiaf300_supplementary_data.zip › Figure S13 raw file_pTPPJ_III1_ WUS probe_compet_mut compet.tif]

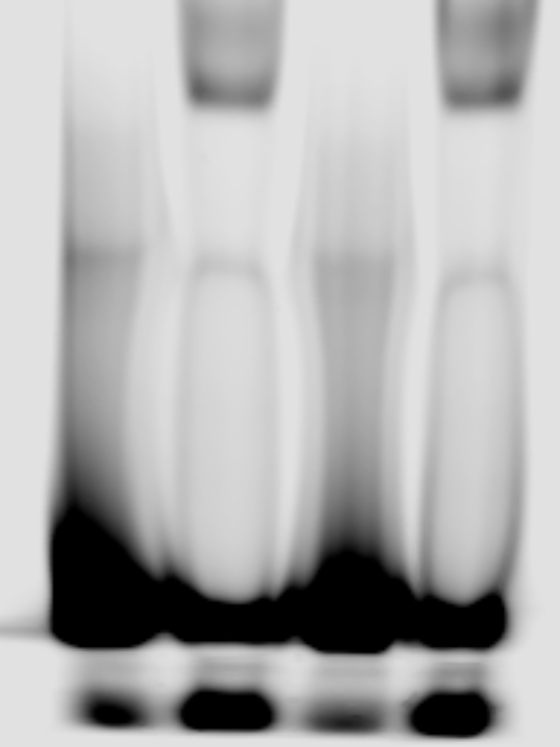

Supplement: kiaf300_Supplementary_Data [file kiaf300_supplementary_data.zip › Figure S13 raw file_pTPPJ_III2_ WUS probe_compet_mut compet.tif]

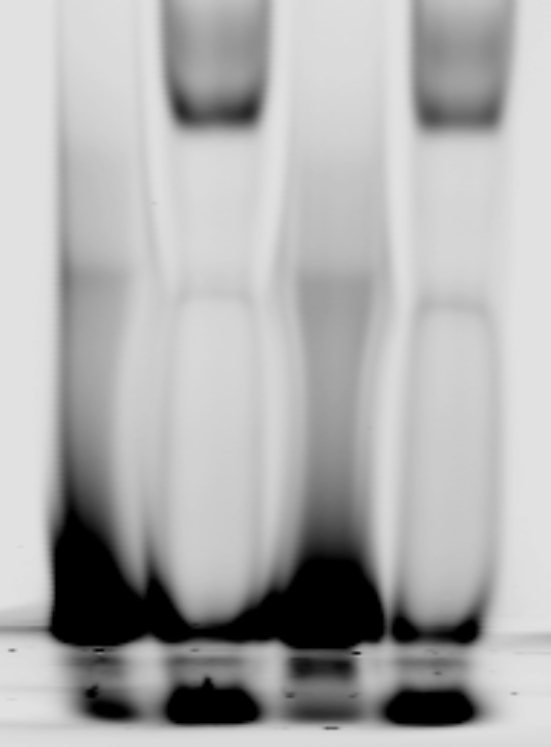

Supplement: kiaf300_Supplementary_Data [file kiaf300_supplementary_data.zip › Figure S13 raw file_pTPPJ_III3_ WUS probe_compet_mut compet.tif]

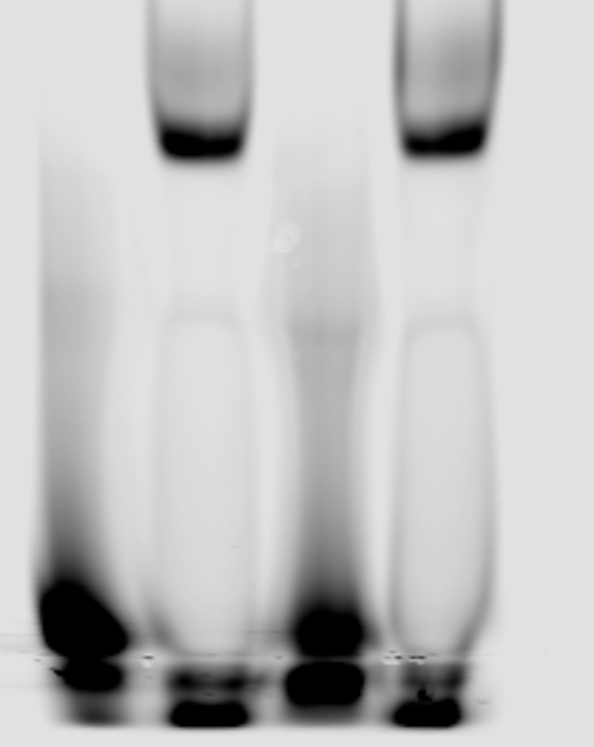

Supplement: kiaf300_Supplementary_Data [file kiaf300_supplementary_data.zip › Figure S13 raw file_pTPPJ_I_WUS probe_compet_mut compet.tif]

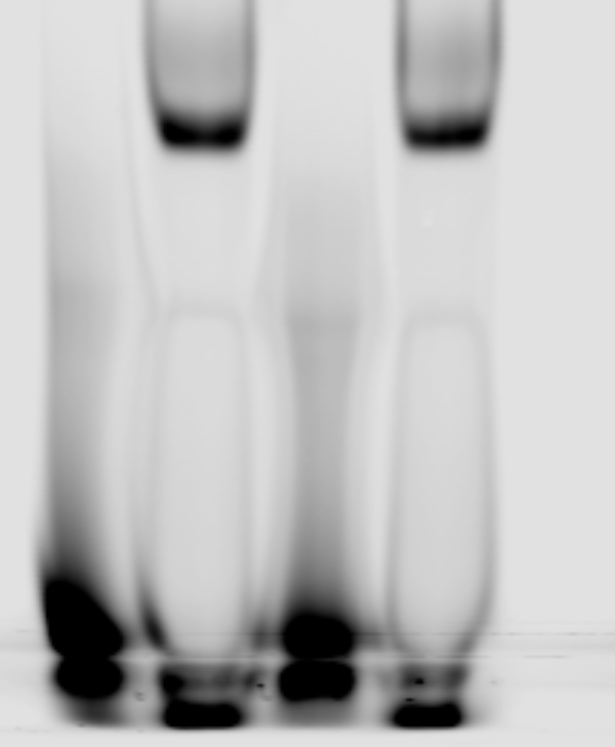

Supplement: kiaf300_Supplementary_Data [file kiaf300_supplementary_data.zip › Figure S13 raw file_pTPPJ_II1_WUS probe_compet_mut compet.tif]

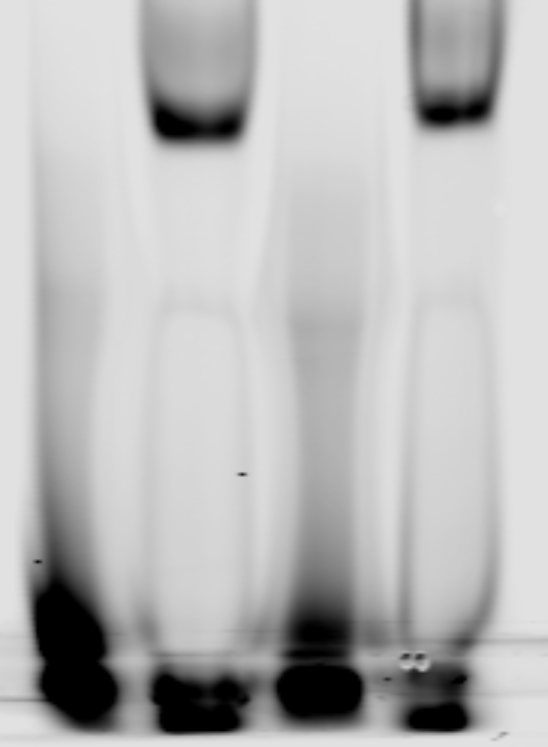

Supplement: kiaf300_Supplementary_Data [file kiaf300_supplementary_data.zip › Figure S13 raw file_pTPPJ_II2_WUS probe_compet_mut compet.tif]

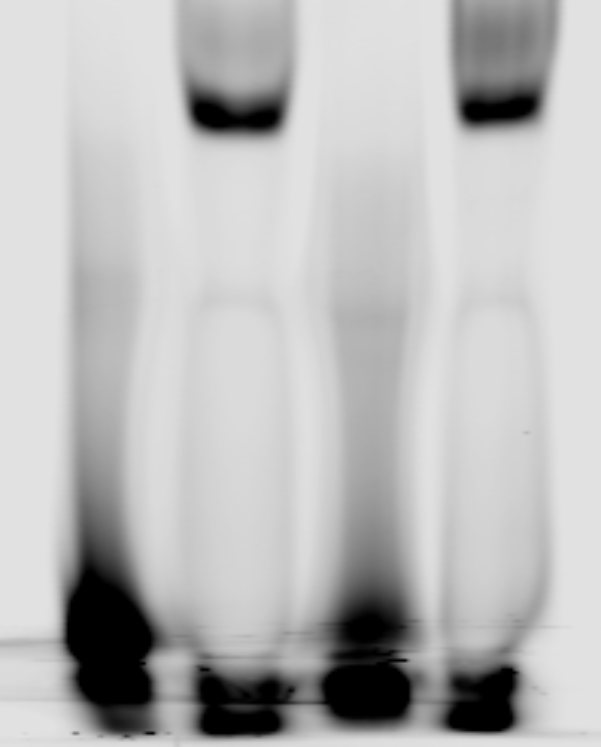

Supplement: kiaf300_Supplementary_Data [file kiaf300_supplementary_data.zip › Figure S13 raw file_pTPPJ_II34_ WUS probe_compet_mut compet.tif]
